# Structural Mechanism of ω-Currents in a Mutated Kv7.2 Voltage Sensor Domain from Molecular Dynamics Simulations

**DOI:** 10.1021/acs.jcim.0c01407

**Published:** 2021-02-11

**Authors:** Giulio Alberini, Fabio Benfenati, Luca Maragliano

**Affiliations:** † Center for Synaptic Neuroscience and Technology (NSYN@UniGe), Istituto Italiano di Tecnologia, Largo Rosanna Benzi, 10, 16132 Genova, Italy; ‡ Department of Experimental Medicine, Università degli Studi di Genova, Viale Benedetto XV, 3, 16132 Genova, Italy; ∥ IRCCS Ospedale Policlinico San Martino, Largo Rosanna Benzi, 10, 16132 Genova, Italy; § Department of Life and Environmental Sciences, Polytechnic University of Marche, Via Brecce Bianche, 60131 Ancona, Italy

## Abstract

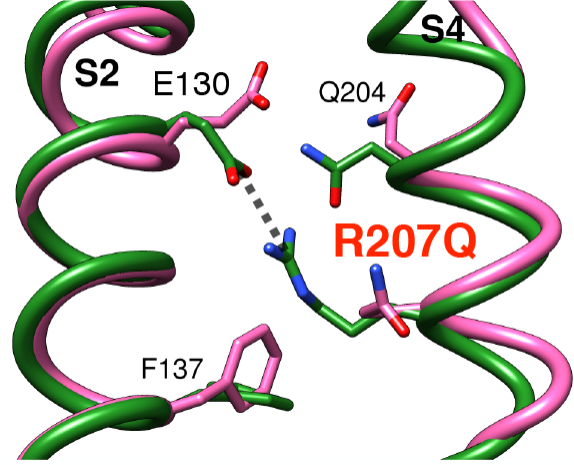

Activation of voltage-gated ion channels is regulated by conformational changes of the voltage sensor domains (VSDs), four water- and ion-impermeable modules peripheral to the central, permeable pore domain. Anomalous currents, defined as ω-currents, have been recorded in response to mutations of residues on the VSD S4 helix and associated with ion fluxes through the VSDs. In humans, gene defects in the potassium channel Kv7.2 result in a broad range of epileptic disorders, from benign neonatal seizures to severe epileptic encephalopathies. Experimental evidence suggests that the R207Q mutation in S4, associated with peripheral nerve hyperexcitability, induces ω-currents at depolarized potentials, but the fine structural details are still elusive. In this work, we use atom-detailed molecular dynamics simulations and a refined model structure of the Kv7.2 VSD in the active conformation in a membrane/water environment to study the effect of R207Q and four additional mutations of proven clinical importance. Our results demonstrate that the R207Q mutant shows the most pronounced increase of hydration in the internal VSD cavity, a feature favoring the occurrence of ω-currents. Free energy and kinetics calculations of sodium permeation through the native and mutated VSD indicate as more favorable the formation of a cationic current in the latter. Overall, our simulations establish a mechanistic linkage between genetic variations and their physiological outcome, by providing a computational description that includes both thermodynamic and kinetic features of ion permeation associated with ω-currents.

## Introduction

Activation of ion conductance in voltage-gated ion channels (VGICs) is regulated by four peripheral voltage sensing domains (VSDs),^1,2^ each made of four transmembrane helices (S1 to S4) and linked to the central pore domain (PD).^3−8^ In response to variations of the transmembrane potential, positively charged amino acids on the S4 helix (typically arginines) change position, inducing a conformational transition in the VSD^9,10^ that is transmitted to the PD and triggers its opening to ion translocation.^11−16^ The movement of the charged residues also generates an electric current called the gating current.^17^

In functional channels, conduction through the VSD is impeded by a hydrophobic constriction separating the extracellular from the intracellular space, and neither water nor ions can pass through it. Mutations in the VSD can result in an altered response to transmembrane voltage and changes in channel gating, a common mechanism of genetically inherited channelopathies.^18−24^ When mutations affect the S4 arginines, anomalous currents (named gating pore currents, ω-pore currents, or ω-currents) have been recorded for various channels^25−27^ and attributed to protons^28,29^ or cations^30−32^ passing directly through the VSD. In addition to their pathophysiological relevance, ω-currents have also been exploited to dissect the structural features of VSDs,^33,34^ since they are related to mutations of residues that stabilize the sensor in the different active or resting states. The human gene KCNQ2 encodes the Kv7.2 subunit of voltage-gated K^+^ channels,^35,36^ and its mutations are associated with neurological disorders in newborns characterized by a wide phenotypic heterogeneity,^37^ including benign familial neonatal seizures (BFNS) and peripheral nerve hyperexcitability (PNH).^38^ In particular, studies on a patient affected by PNH^39^ have identified a Kv7.2 mutation corresponding to the loss of the R207 arginine in S4 (R207Q). Electrophysiological studies revealed a positive shift in the voltage dependence of activation^40^ and the onset of an ω-current at depolarized potentials^41^ in the mutant and suggested that neutralizing the R207 charge alters the stability of the open state (active) VSD configuration by breaking a pivotal interhelix salt bridge. Specifically, Taglialatela and collaborators^41^ performed macroscopic and gating current measurements in Kv7.4, a channel highly related to Kv7.2 both functionally and structurally, illustrating the presence of an ω-current in the R207Q mutant that was associated with an inward Na^+^ or proton flux capable of providing a persistent depolarizing force. Other Kv7.2 pathogenic mutations (R213W, R213Q, D212G, E140Q) were described to impair the channel functional activity, supposedly by affecting the stability of the VSD active state.^24,41^ Moreover, those involving S4 residues (R213W, R213Q, D212G) did not cause activation of ω-currents.^41^ The two mutations involving R213 have been found in children with two distinct pathologies: R213W induces BFNS,^42^ while R213Q produces neonatal epileptic encephalopathy with severe neurocognitive delay.^43^ The D212G mutation, affecting the Kv7 conserved negatively charged residue between the arginine residues R210 and R213, was identified in a BFNS case.^19^ The fourth mutation, located in the S2 helix (E140Q) was recently observed in a patient with neonatal onset developmental and epileptic encephalopathies (DEE).^24^ To unravel the structural features underlying such diverse effects, we resorted to use of atomistic molecular dynamics (MD) simulations combined with free energy (or potential of mean force, PMF) and kinetics calculations. MD simulations have proven useful to study gating pore currents in different channels. Unbiased simulations were employed to determine the role of specific residues in regulating VSD hydration in sodium^44,45^ and calcium^46^ channels, while a constant electric field was applied to mimic the action of a potential gradient across the membrane to induce ω-currents in potassium^34,47,48^ and proton^49^ channels. More recently, free energy calculations were performed to illustrate the mechanism of ionic permeation through mutated VSDs of sodium^50^ and proton^51,52^ channels. However, genotype–phenotype correlations are still limited and need further detailed inspection for the various VGICs. Here, we first generated a homology model of the Kv7.2 VSD, based on the structure of the close homologue Kv7.1 channel^53^ (PDB ID 5VMS). We refined the model in a water/membrane environment using extensive MD simulations, and then we modified the native sequence by introducing the mutations described above. A cryo-EM structure of the human channel with the VSDs in the active state has recently been published.^54^ Unrestrained MD simulations and a comparison with our model show that the latter recapitulates all the relevant features of the experimentally determined VSD conformation. Our unbiased MD simulations reveal that most of the mutations affect the stability of the active-state structure, but only in the case of the R207Q mutation an uninterrupted water wire (defined as a sequence of contiguous water molecules occupying a restricted region of space) is observed within the transmembrane sensor domain. To further investigate the link between the enhanced hydration pattern and the formation of an ω-current, we used the Voronoi tessellated Markovian milestoning (VTMM) method^55^ with *soft*-walls restraining potentials^56^ to calculate the thermodynamic and kinetic features of sodium permeation through the wild-type (WT) channel and the R207Q mutant. In the WT structure, the highly conserved interhelix salt bridge between R207 and E130 prevents the passage of water molecules through the hydrophobic constriction, thus resulting in an extremely high energy barrier for the passage of a sodium ion. Conversely, in the R207Q mutant, the formation of the continuous water wire facilitates sodium permeation, significantly lowering the barrier. The absence of the gating charge in the 204 position in the Kv7.2 structure, where a glutamine is found (Q204), helps in the formation of the water pore, thus contributing to the cationic leak. Globally, our results describe a clear picture of Na^+^ permeation in R207Q, responsible for the formation of the ω-current, and quantify the effect of the mutation on the functional destabilization of the Kv7.2 VSD. Our approach, providing an estimation of both thermodynamic and kinetic features related to ω-currents could be useful as a proof-of-concept for future applications aimed at establishing a mechanistic linkage between a genetic variation and its physiological outcome.

## Materials and Methods

### MD Simulations Set Up

For all MD simulations described in this work, we used the NAMD software^57^ (version 2.12) and the CHARMM36m^58−60^/CHARMM36^61^ parameters for the protein and lipids, respectively, together with the associated ionic parameters with NBFIX corrections.^62−64^ The systems were simulated in the NPT ensemble using the Nosé–Hoover Langevin piston method^65,66^ to maintain the pressure at 1 atm. The oscillation period of the piston was set at 50 fs, and the damping time scale at 25 fs. The Langevin thermostat was employed to maintain the temperature at 310 K with a damping coefficient of 1 ps^–1^. Tetragonal periodic boundary conditions (PBCs) were applied to the simulation box to remove surface effects. Long range electrostatic interactions were calculated using the particle mesh Ewald (PME) algorithm,^67^ with a spline interpolation order 6 and a maximum space between grid points of 1.0 Å. Short range electrostatic and van der Waals interactions were calculated with a cutoff of 12 Å and the application of a smoothing decay starting to take effect at 10 Å. A time step of 2 fs was employed. To ensure maximum accuracy, electrostatic and van der Waals interactions were computed at each simulation step. All covalent bonds involving hydrogen atoms were kept fixed using the SHAKE/SETTLE algorithms.^68,69^ All MD trajectories were visualized and analyzed using UCSF Chimera^70^ and VMD^71^ with Tcl scripts.

### Structural Modeling of the Kv7.2 VSD

We used homology-based modeling to build five different structures of the human Kv7.2 (hKv7.2) VSD in an activated, depolarized configuration, starting from four different templates. A thorough description of the modeling and refinement strategy and of the procedure followed to choose the best model from the pool is provided in the Supporting Information. Briefly, the templates were (1) the mammalian Kv1.2^72^ (used for two models by employing two different approaches, as described below), (2) a previously refined model from the human Kv7.1 (hKv7.1),^73^ (3) the Kv1.2/Kv2.1 chimera,^9^ and (4) the Kv7.1 channel from *Xenopus laevis* (xKv7.1, PDB ID 5VMS,^53^ 3.7 Å   resolution). The SWISS MODEL server^74^ was used to generate all models except for a second one from the first template, which was built with MODELLER.^70,75^ We refined all five structures via short MD simulations in an explicit membrane–solvent environment. The proteins were embedded into a heterogeneous lipid patch made of a mixture of 1-palmitoyl-2-oleoyl-*sn*-glycero-3-phosphocholine (POPC) and cholesterol, a heterogeneous environment already adopted to study the ionic permeation of other membrane proteins.^46,76,77^ In our case, the lipid environment is formed from ∼90% POPC lipids and ∼10% cholesterol in each leaflet. Experimental works have demonstrated the role of a minor lipid of the inner membrane leaflet, phosphatidylinositol 4,5-bisphosphate (PIP2), as a cofactor in Kv7 channel activation, promoting coupling of VSDs to pore domains (PDs).^78^ In particular, the VSD S2–S3 loop and S4–S5 linker are considered important hot spots for PIP2 binding at the cytoplasmic side of the channel.^79−82^ For this reason, we inserted two PIP2 molecules in the inner leaflet, thus mimicking its typical low concentration (∼1%) and partitioning in mammalian cells.^81^

The membrane and proteins were then solvated with explicit TIP3P water molecules,^83^ and the total charge was neutralized with a 150 mM NaCl solution. In all systems, the thickness of water layers on top and bottom of the bilayer and the protein was set to 25 Å and the starting size was approximately [90 × 90 × 105] Å^3^, which ensured a distance larger than 20 Å between adjacent images of the protein during the simulations. We extended by 30 ns the CHARMM-GUI default equilibration procedure, in which short runs are performed first with restraints on all the protein and lipid heavy atoms, which then are gradually released. Then, for each model, we performed an unrestrained run of 10 ns in the NPT ensemble.

To evaluate the quality of the models, we monitored the presence and persistence of the salt bridge interactions between the side chains of the S4 arginine residues and the facing negatively charged side chains on S1–S3 helices, known to stabilize the active site of VSDs,^9,21,22,40,41,53,84^ as described in Supporting Information. Based on these criteria, we selected as best model the one built from the xKv7.1 cryo-EM structure and used it in all the simulations reported in this work. The xKv7.1 channel shows about 60% sequence similarity with hKv7.2, both globally and in the VSD region.^85,86^ The template structure corresponds to a so-called decoupled state at depolarized potential, with a closed pore and the VSDs in an activated conformation, which is the configuration associated with the electrophysiological effects observed for the R207Q mutation in ref (41).

A remark concerning residue naming is also necessary. Charged residues of VGIC VSDs are usually indicated by a simplified enumeration to facilitate comparison between different structures. Channels of the Kv7 family have a missing arginine in the middle of the S4 helix that causes a gap in the numbering and generated different adopted solutions. Here, following the notation presented in refs (21 and 41), we associate names R1, R2, Q3, and R4–R6 with R198, R201, Q204, R207, R210, R213, respectively, and so, where needed, we indicate the R207Q mutation as R4Q. Other residues names used in this notation are E1 for E130, E2 for E140, D1 for D172, D2 for D212, and R* for R144. The extended residue numbering is employed hereafter, while the shortest one is used in the Supporting Information to compare different Kv7 VSD structures.

### MD Simulations of the Optimal Model for WT and Mutant VSD

Once we selected the optimal model as described, the system was further equilibrated for an additional 30 ns, continuing from the extended CHARMM-GUI protocol described above, to allow proper hydration of solvent-exposed regions of the VSD. In the latter equilibration stage, we applied soft restraints only on the Cα atoms of the most external residues on both sides of the α helices (i.e., S1 residues 93–97 and 108–112, S2 residues 123–127 and 144–148, S3 residues 167–171 and 181–185, and S4 residues 195–199 and 212–219) with a force constant of 1 kcal/(mol·Å^2^). To evaluate the effect of membrane composition on VSD conformations, we also built a second system from the same optimal VSD model, with the protein embedded in a homogenoeus POPC patch. The same extended equilibration protocol was followed. We name the two systems HET and HOM, respectively. After the equilibration stage, a 210 ns long unrestrained production run was carried out for both systems. MD simulation parameters were the same as described above.

By using a configuration of the WT HET system extracted from the extended equilibration mentioned above, five mutant VSDs were prepared (R207Q, R213W, R213Q, D212G, and E140Q). Coordinates for the atoms of the mutated residues were obtained according to the Dunbrack rotamer library.^87^ For each mutant, we performed an additional 10 ns of equilibration with soft restraints on the external Cα atoms only, and finally a 500 ns long unrestrained MD run. MD simulation parameters were the same described above.

### Structural Analysis

We monitored the stability of the salt bridges known to stabilize the activated state of the VSD^9,21,22,40,41,53,84^ by measuring the distance between the last carbon atoms of the residue side chains to take into account rotations of the charged groups. Specifically, we introduced the following distances (we indicate specific atoms by the one-letter code and number of the residue, the atom name, and the helix name in superscript):

- R207CZ^S4^–E130CD^S2^, named *d*1
- Q204CD^S4^–E130CD^S2^, named *d*2
- R210CZ^S4^–E140CD^S2^, named *d*3
- R213CZ^S4^–D172CG^S3^, named *d*4

Although the distance *d*2 is not associated with a SB, we found it useful to check the orientation of Q204 as well. To monitor the stability of the overall VSD structure along the simulated trajectories, we also calculated the following cross-distances, that is, distances between Cα atoms of facing residues on opposite helices of the domain:

- V99CA^S1^–D172CA^S3^, named cd1
- V103CA^S1^–V175CA^S3^, named cd2
- S110CA^S1^–V182CA^S3^, named cd3
- Y141CA^S2^–M211CA^S4^, named cd4
- I134CA^S2^–L203CA^S4^, named cd5

### VSD Hydration

Water occupancy in the internal VSD cavity was quantified in two ways as described in Supporting Information, using a selection based on water position along the VSD axis in a 10 Å interval centered around the hydrophobic constriction site (HCS) (*axis* selection) or within a sphere of radius 5 Å around the HCS (*sphere* selection). Water density profiles along the VSD axis were computed for the WT and the R207Q sensors from 210 ns of unrestrained trajectories of the two systems, averaging over 210 consecutive configurations extracted every 1 ns.

## Computational Methods

### Thermodynamic and Kinetic Calculations

A still growing family of algorithms to calculate the kinetic properties of (bio)chemical processes originates from the milestoning method.^88^ In the initial approach,^89^ the dynamical features of a reactive event are reconstructed out of a set of short trajectories freely evolving between a set of hypersurfaces (named the *milestones*), placed sequentially along the reaction path between the reactant and the product states. The method was employed to investigate many biophysical processes such as protein translocations,^90,91^ membrane permeation,^92,93^ or conformational changes and substrate binding in enzymatic reactions.^94−96^

Here, we use an alternative version known as Voronoi tessellated Markovian milestoning (VTMM),^55,56^ where the milestones are identified as being the edges of the cells of a complete Voronoi tessellation of collective variable (CV) space. In more detail, if we consider a set of *M* points in the CV space (*z*_1_, *z*_2_, ..., *z*_*M*_), we can associate a partition of the space in *M* Voronoi cells (*B*_1_, *B*_2_, ..., *B*_*M*_) with them, each one being defined as the region where each other point is closer to *z*_α_ than to any other *z*_β_ of the set. It was demonstrated in ref (55) that the statistical features of the longtime dynamics of the system can be reconstructed from independent simulations, each confined within one Voronoi cell, as long as the confinement is not perturbing the dynamical properties of the system in the interior of the cell and the probability flux in and out of it. Such a confinement can be realized by using velocity reflections at the cell boundaries (*hard*-walls VTMM^55^) or via the introduction of half-pseudoharmonic restraining potentials (*soft*-walls).^56^ The latter approach is simpler to realize when using highly optimized MD codes. Although it requires that the portions of the trajectory that are transiently out of the cells are omitted from the analysis, these are minimized by proper tuning of the restraint parameter. In the simple case of a 1D CV used here, that is, the position of the ion along the VSD main axis *z*, the *soft*-walls potential in the Voronoi cell *B*_α_ to be added to the potential energy described by the CHARMM force field is
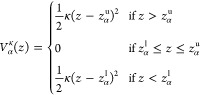
1where *z*_α_^u^ and *z*_α_^l^ denote the positions of the upper and lower edges of the cell *B*_α_, respectively, and in our case are the midpoints between the center *z*_α_ and the adjacent ones.

The equilibrium probability of finding the system in cell *B*_α_, which we indicate as π_α_, can be obtained by imposing a balance equation, that is, by requiring that the net probability flux in and out of each cell is zero.^55^ From π_α_, the free energy associated with the same cell is calculated as *F*_α_ = −*k*_B_*T* ln π_α_, with *k*_B_ the Boltzmann constant and *T* the temperature of the system. By considering the edges of the Voronoi cells as milestones, the dynamics of the system can then be reduced to that of a discrete-state/continuous-time Markov chain in the state space of milestone indices.^97^ This implies defining a rate matrix *q*_*ij*_ for the full process, with *i* and *j* indices of milestones, whose elements can be obtained from the individual simulations in the different cells.^55^ Specifically, *q*_*ij*_ = *N*_*ij*_/*R*_*i*_, where *N*_*ij*_ = ∑_α=1_^*M*^π_α_(*N*_*ij*_^α^/*T*_α_) and *R*_*i*_ = ∑_α=1_^*M*^π_α_(*R*_*i*_^α^/*T*_α_), with *N*_*ij*_^α^ being the number of transitions from *i* to *j* in cell *B*_α_, *R*_*i*_^α^ being the total time that milestone *i* was the last crossed in cell *B*_α_, and *T*_α_ being the duration of the simulation in the cell. The rate matrix *q*_*ij*_ is then used to calculate the mean first passage times (MFPTs) from any milestone to any other. For instance, if τ_*i*_^*K*^, with *i* = 1, ..., *K* – 1, are the MFPTs from milestone *i* to milestone *K* (τ_*K*_^*K*^ = 0, by definition), then these MFPTs can be obtained by solving the linear system of equations ∑_*j*=1_^*K*–1^*q*_*ij*_τ_*j*_^*K*^ = −1,with *i* = 1, ..., *K* – 1. Previous applications of VTMM include ligand binding,^98,99^ nucleation from an ionic liquid,^100^ and ion translocation in proteins.^101,102^ Here, we computed the MFPTs from all milestones, starting with the most extracellular one, to the last one at the intracellular side, thus representing an ion current flowing into the cell, as experimentally observed for the Kv7.2 R4Q mutant.^41^ Since we are interested in the relative permeation in the mutant versus the wild-type VSD and because there is only one entry portal and one exit portal in the protein structure, we neglect the entry kinetics contributions related to bulk concentration and diffusivity that are discussed in ref (98).

### System Setup for VTMM-MD Simulations

To generate configurations of the Na^+^ ion inside the cavity of the WT and the R207Q mutant VSD, we used temperature accelerated molecular dynamics (TAMD)^103^ and steered molecular dynamics (SMD).^104^ A description of these methods and details of the simulations are provided in the Supporting Information. The VSD *z*-axis was then discretized in a grid of 84 cells, each 0.67 Å wide. The width of milestoning cells must be such that in each restricted trajectory one has sufficient sampling of the CV space, which in turn amount to differences of only few kcal/mol between adjacent cells. Since we anticipated large free energy barriers for the permeation of Na^+^ through the VSDs, we chose closely spaced cells and accrued statistics over 50–60 ns of simulation. The starting configuration in each cell obtained from the preparatory TAMD/SMD simulations was minimized for 1000 steps and, after heating up gradually back to 310 K, equilibrated for about 200 ps with protein restraints. Then, in each cell, production was performed for 50–60 ns until convergence of the quantities *F*_α_, *N*_*ij*_^α^, and *R*_*i*_^α^ defined above. To avoid any rigid body rotational or translational displacement of the protein, soft harmonic restraints were maintained on the Cα atoms of the apical residues of each α helix (S1 residues 93–97 and 108–112, S2 residues 123–127 and 144–148, S3 residues 167–171 and 181–185, and S4 residues 195–199 and 212–219). In each run, the Na^+^ ion was confined in the cell with the application of half-harmonic restraints (force constant 100 kcal/(mol·Å^2^)) at the upper and lower boundaries of the *z* variable, which is the reaction coordinate of the process. An additional so-called flat-bottom cylindrical restraint acting on the Na^+^ ion only, restricting its motion orthogonally to the *z* axis, was added to prevent its lateral escape from the VSD. The cylinder is oriented along the *z* axis and has a 20 Å diameter, while the force constant is 100 kcal/(mol·Å^2^). The restraining potential is zero inside the cylinder and acts only when its border is crossed, so given the diameter, it does not prevent protein–ion interactions. The COLVAR module^105^ was used to introduce these restraints, as done in our previous work.^101^ All milestoning simulations required a cumulative time of ∼9.6 μs.

Na^+^ coordination was calculated by counting the number of water, protein, and lipid heavy atoms within the first Na^+^ solvation shell (cutoff distance 3 Å).

**Figure 1:**
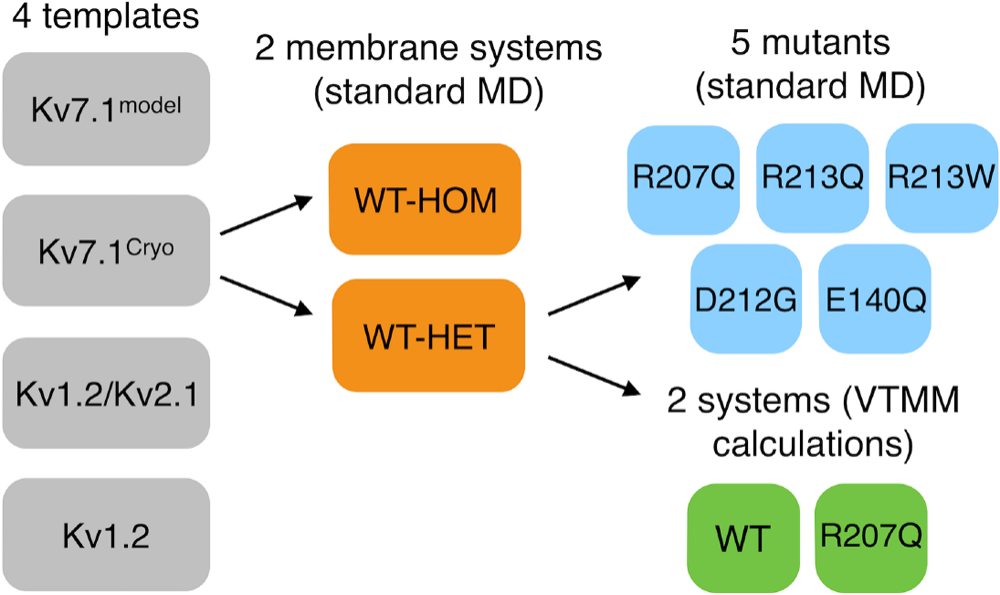
Worflow of the modeling and simulations performed. PDB codes of templates are 5VMS (Kv7.1^Cryo^), 2R9R (Kv1.2/Kv2.1), and 2A79 (Kv1.2).

We conclude this section with a summary of the simulations performed in this work in Figure 1 and Table 1.

**Table 1:** Summary of All the MD Simulations of hKv7.2 Performed in This Work^a^

system	method	simulation length (ns)
WT (HOM + HET)	MD	210 + 210
R207Q	MD	500
R213W	MD	500
R213Q	MD	500
D212G	MD	500
E140Q	MD	500
WT and R207Q	VTMM-MD	9660 (cumulative)
WT (PDB ID 7CR0)	MD	200

a Cumulative time: ∼12800 ns.

## Results

### Standard MD Simulations of WT hKv7.2 VSD and Its Mutants

We performed unrestrained MD simulations of the WT VSD and of each mutant in explicit membrane–water environment.

### MD Simulations of the WT hKv7.2 VSD Model

As described in Materials and Methods, we generated five models starting from four different templates and selected the one that showed the higher degree of persistence of active-state stabilizing residue–residue interactions.

**Figure 2:**
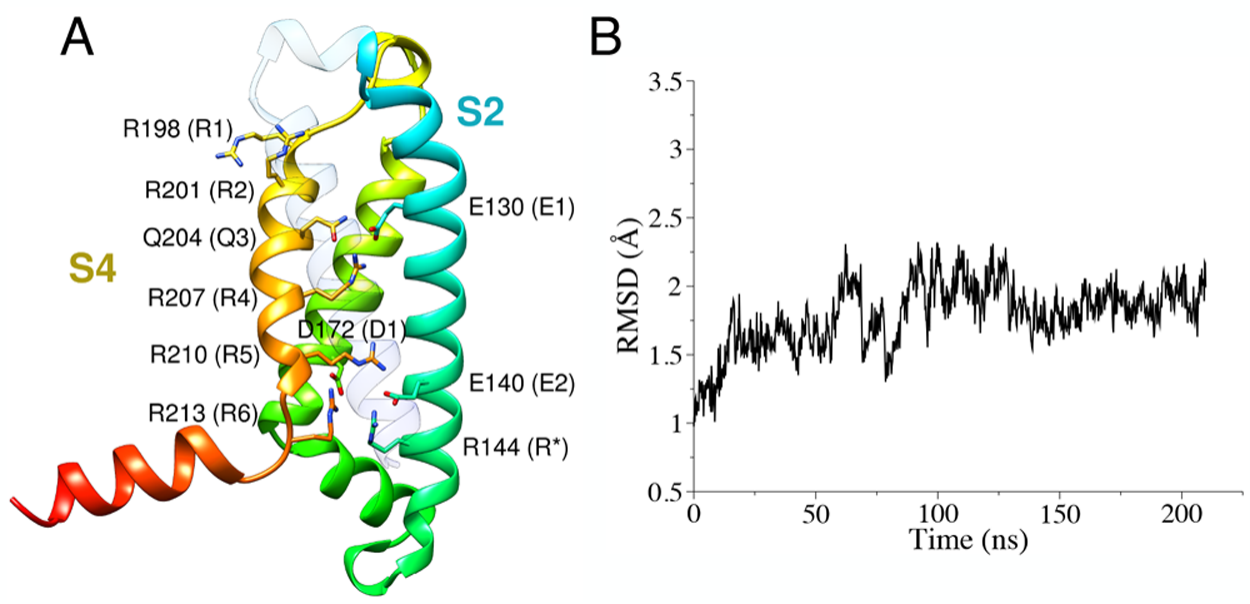
(A) hKv7.2 VSD structural model, represented as ribbons colored from blue (N-terminus) to green to red (C-terminus). The S1 helix is left transparent for clarity. Side chains of relevant residues are represented as sticks. (B) Backbone RMSD values of the TM α-helices for the VSD model in the heterogeneous membrane.

**Figure 3:**
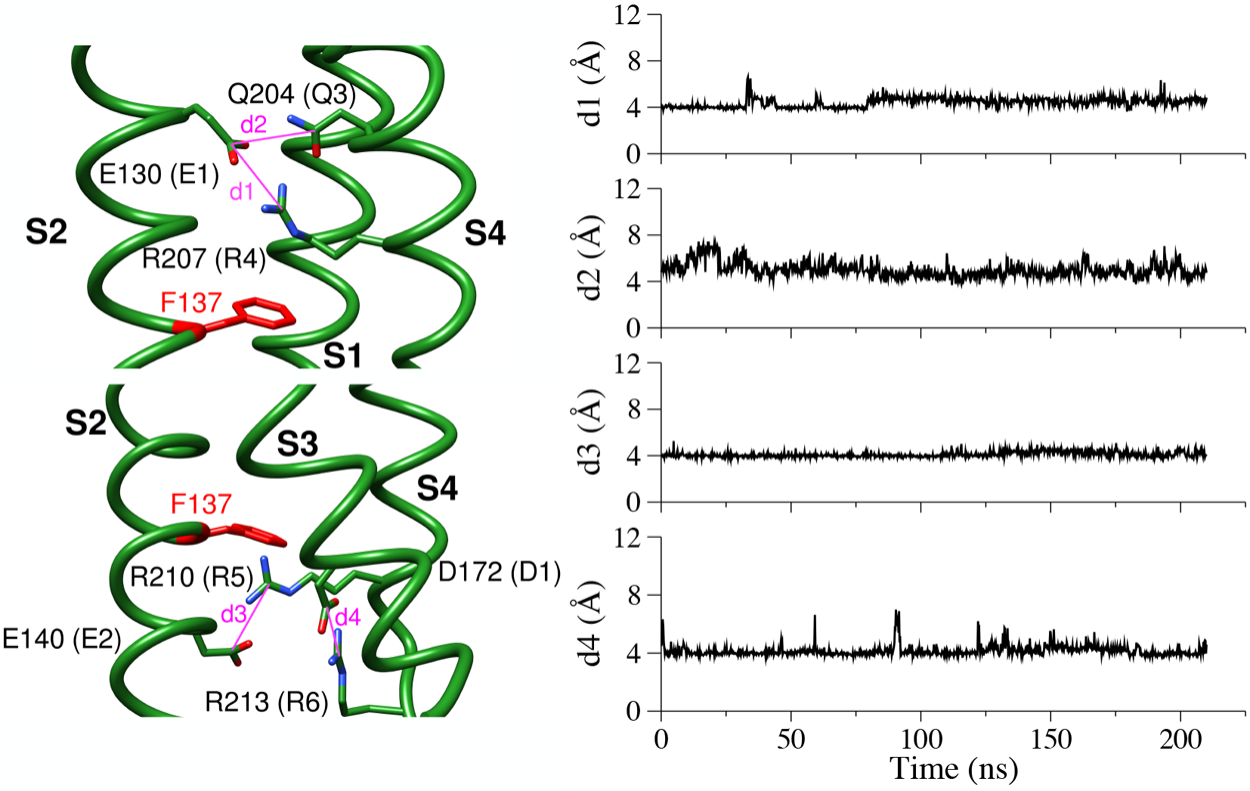
Time-evolution of the *d*1–*d*4 distances for the WT, hKv7.2 VSD in the heterogeneous membrane.

Our optimal protein model is shown in Figure 2A and in Figure S1 in its membrane–water environment. MD runs were performed to allow protein relaxation into two different hydrated lipid environments (named HET and HOM, respectively, Figure S2). The root-mean-squared deviation (RMSD) of the protein backbone of the four transmembrane (TM) α-helices from the initial conformation was used as an index of stability. A plateau of ∼2–2.25 Å  was observed for both systems (Figures 2B and S3 for the HET and HOM systems, respectively). Furthermore, the analysis of the *d*1–*d*4 distances describes stable SBs in the upper and lower vestibule of the VSD (Figures 3 and S4 for the HET and HOM systems, respectively, and Table S1). The stationarity of these interactions demonstrates that the model preserves its active configuration along the MD trajectories.

During the MD runs, water molecules hydrate the internal cavity of the VSD only in the upper and lower vestibule. In particular, the equilibrated configurations of the two systems are characterized by a low hydration in the HCS. To quantify water content, we calculated the number of water molecules in the region with two selections based on the position of water molecules along the VSD axis or within a sphere of 5 Å radius centered in the HCS, as described in Materials and Methods.

**Figure 4:**
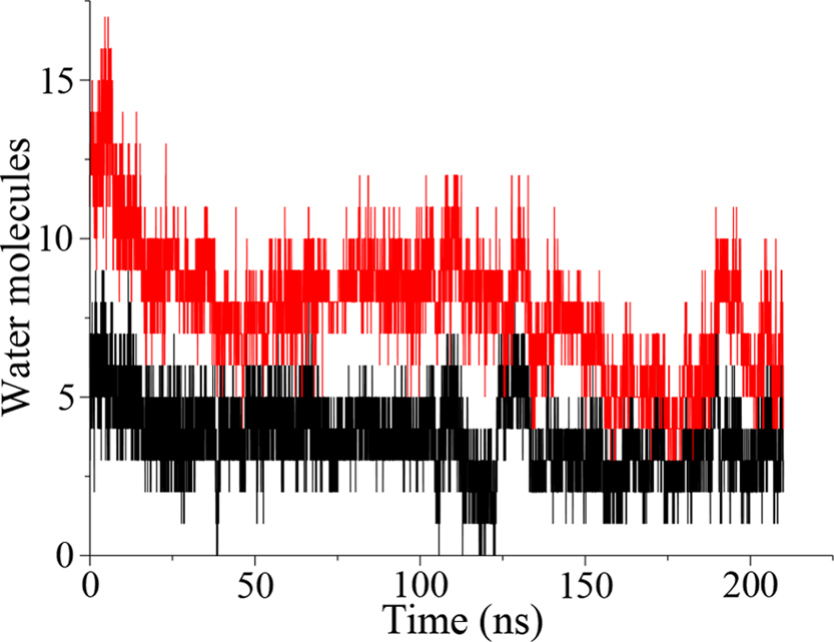
Time-evolution of water occupancy at the HCS in the WT hKv7.2 VSD in the heterogeneous membrane system (red curve, axis selection; black curve, sphere selection).

This analysis shows comparable results for both membrane environments with an almost dry spherical core (average number of water molecules ≤6), as illustrated in Figures 4 and S5 for the HET and HOM systems, respectively.

**Figure 5:**
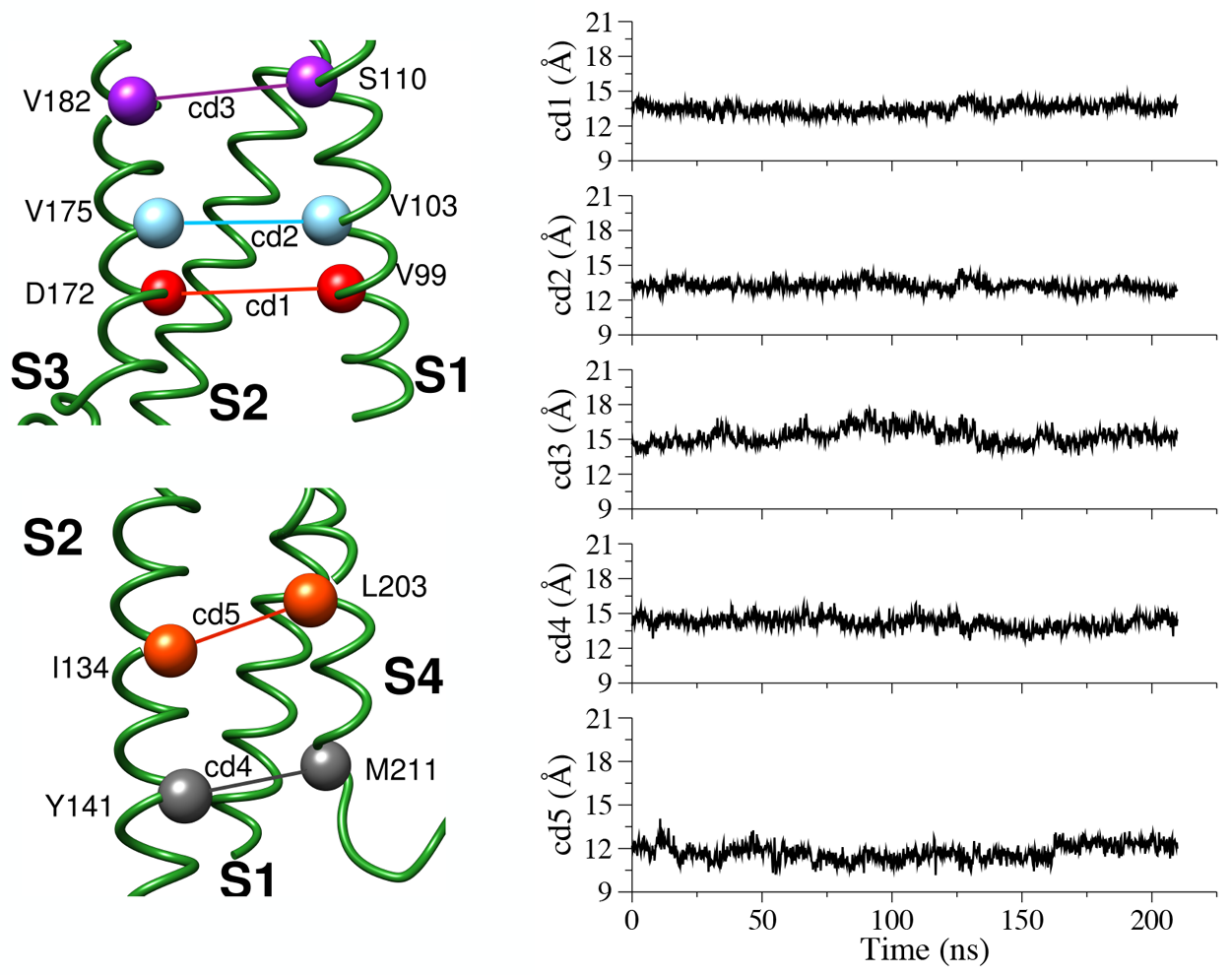
Time evolution of cross-distances in the WT hKv7.2 VSD in the heterogeneous membrane system.

Cross-distances between Cα atoms on facing helices, as defined in Materials and Methods, were used to monitor possible distortions of the protein structure during the MD simulations. Their time evolution along the simulations in both membrane environments show limited variations from the values of the starting configuration for the HET and HOM systems, as described in Figures 5 and S6, respectively, and Table S2. This is in accordance with the RMSD measurements and the modest VSD hydration, which does not alter the initial configuration of the internal cavity.

### MD Simulations of the R207Q hKv7.2 VSD

**Figure 6:**
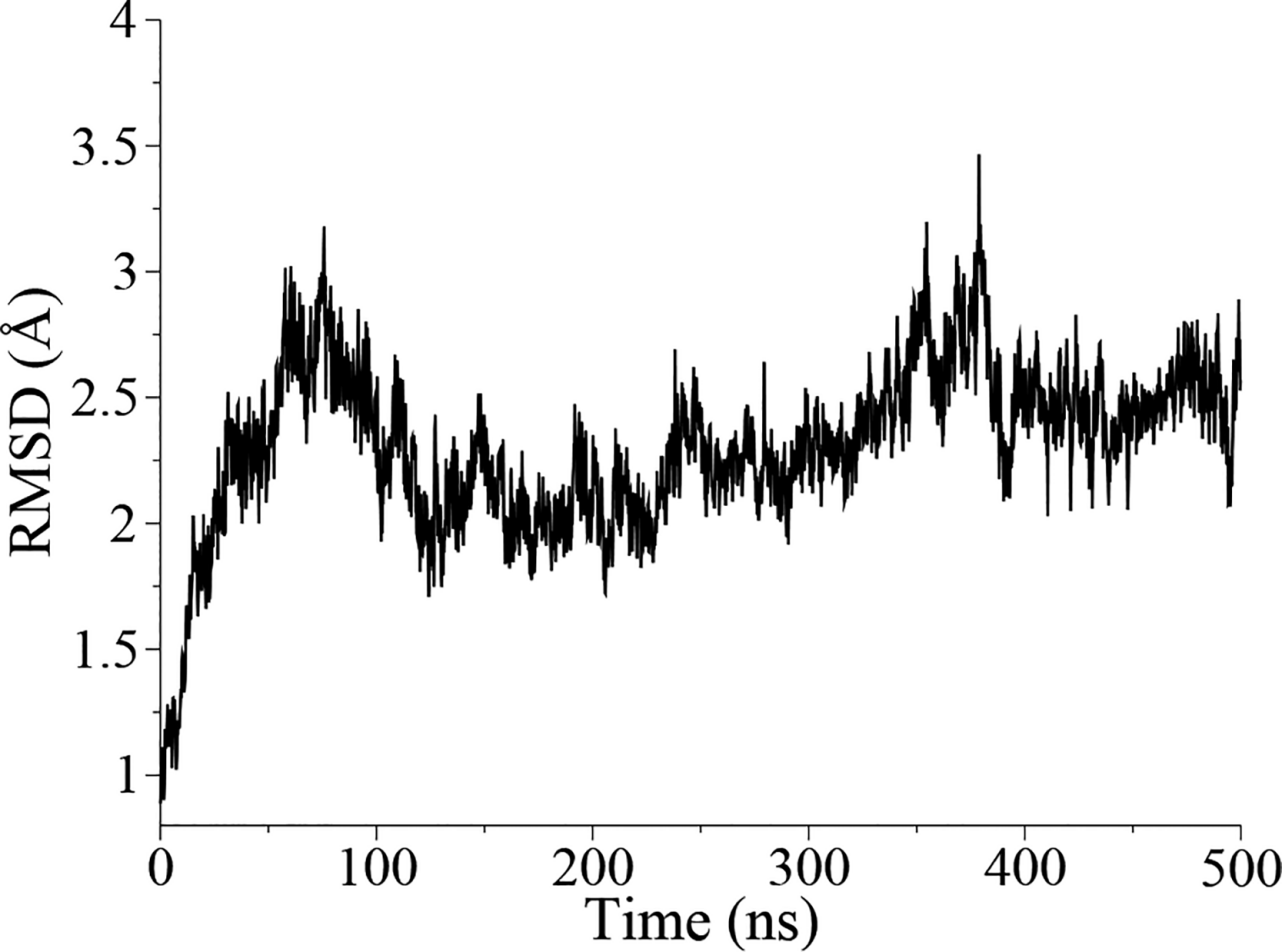
Backbone RMSD values of the TM α-helices for the R207Q hKv7.2 mutant.

**Figure 7:**
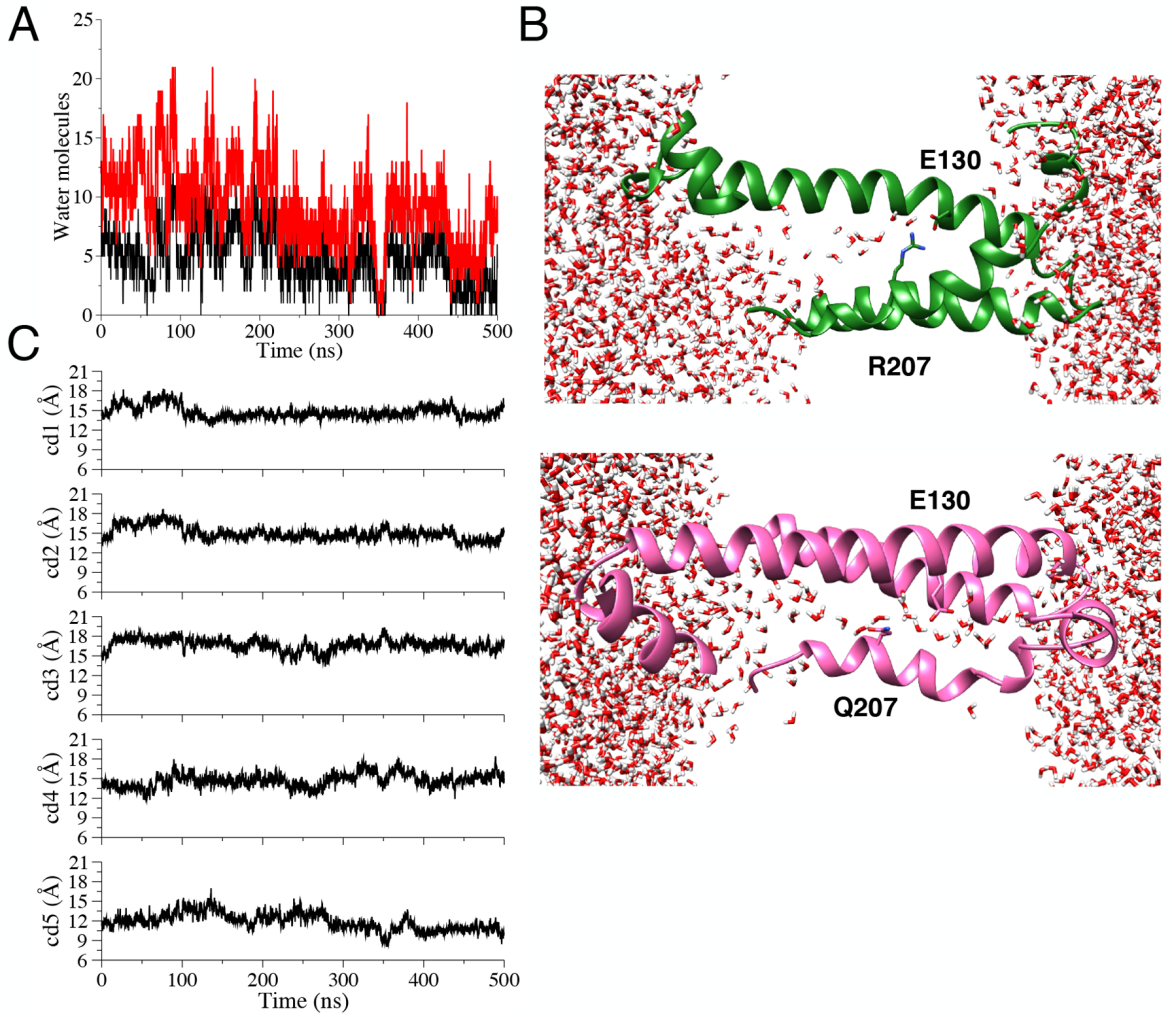
(A) Water occupancy for the R207Q hKv7.2 mutant (red curve, axis selection; black curve, sphere selection). (B) Partial view (helices S1, S2, and S4) of the WT (above, green) and R207Q (below, magenta) VSDs; water molecules and residues are represented as sticks, lipids are omitted for clarity, and the *z* axis is horizontal. (C) Time evolution of cross-distances in the R207Q hKv7.2 mutant.

**Figure 8:**
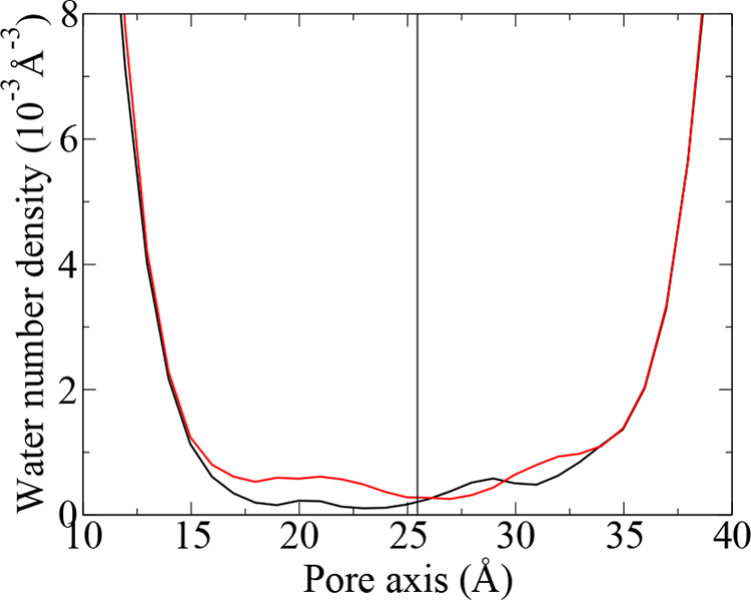
Water density profiles along the VSD axis from the unrestrained simulations of the WT (black) and R207Q mutant (red). The pore axis coordinate increases from the extracellular to the intracellular space. The black line represents the location of the peak of the axial distribution of R207 Cα atom.

For the R207Q system, the MD run was extended up to 500 ns to improve sampling. The time evolution of RMSD values (Figure 6) demonstrates that the global structure is preserved during the run, although more pronounced fluctuations are apparent with respect to the WT system. An increase in hydration is observed at the level of the HCS (Figure 7A), allowing the formation of an uninterrupted water wire across the transmembrane region, as illustrated by snapshots extracted from the simulations in Figure 7B. The increase in HCS hydration in the mutant is coupled to a more pronounced lateral expansion of the helices, as shown by the cd1–cd5 cross-distances (Figure 7C). In Figure 8, we report the water density profiles calculated along the VSD axis from the unrestrained trajectories of the WT and R207Q VSDs (pore axis oriented from the extracellular to the intracellular space). Results show increased hydration in the mutant with respect to the WT in the pore region between 15 and 25 Å, corresponding to the extracellular facing cavity of the VSD and the uppermost side of the HCS, consistent with the simulation snapshots in Figure 7B.

### MD Simulations of Additional hKv7.2 VSD Mutants

We performed unbiased MD simulations of four other mutants (R213W^S4^, R213Q^S4^, D212G^S4^, E140Q^S2^), previously supposed to alter the stability of the activated VSD configuration.^24,41^ The first three involve residues on the S4 helix, but in contrast to R207Q, they are not associated with the onset of ω-currents.^41^ We therefore investigated the molecular mechanism underlying this difference.

The time evolution of RMSD values highlights different degrees of structural stability of the mutated VSDs (Figure S7). In particular, the RMSD profiles of the first three mutants plateau at values slightly higher than the WT, while E140Q seems not to have reached a plateau at all in the time window analyzed. Increased water occupancy of the cavity (Figure S8) and larger fluctuations of cross-distances (Figures S9–S12) are also observed. However, in contrast to what was observed in the case of the R207Q mutant, these mutations do not perturb the SB between the side chains of R207 and E130, as revealed by the time evolution of the *d*1 distance (Figure S13). Hence, in all the additional mutants analyzed, and similarly to the WT system, the presence of this SB forms an obstruction that prevents the hydration pattern observed in the cavity of the R207Q system, a condition that may be associated with the different conduction properties observed in experiments.

### VTMM MD Simulations to Obtain PMF and MFPTs of Na^+^ Permeation

Electrophysiological experiments proved that the Kv7.2 R207Q is associated with the onset of an ω-current^41^ at depolarizing potentials, which was proposed to originate from an inward flux of Na^+^ ions. To verify the hypothesis that R207Q is more permissive to Na^+^ permeation than the WT VSD, we used the VTMM method to calculate the PMF and MFPTs of single-ion translocation through the two proteins.

**Figure 9:**
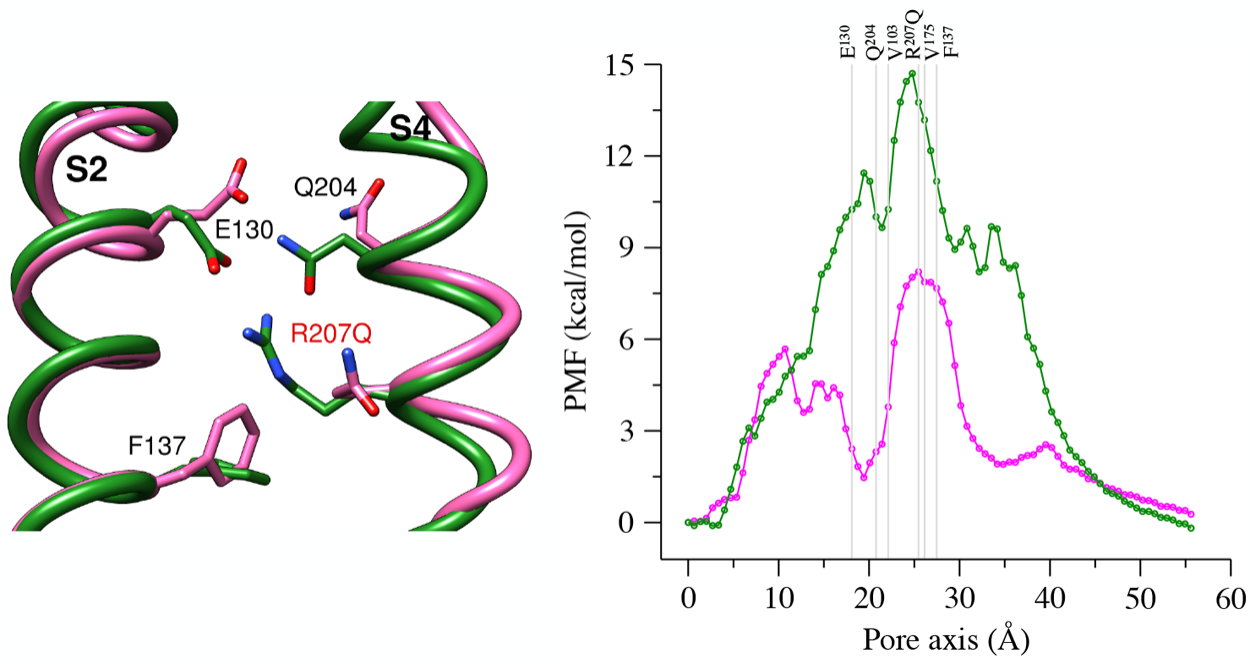
(left) Superposition of the upper vestibule of the WT model (green) and the R207Q mutant (magenta), with relevant residues represented as sticks. (right) PMF profiles for WT (green) and the R207Q (magenta) systems; the pore axis is oriented from the extracellular to the intracellular space, and the positions of the Cα atoms of relevant residues are represented as gray lines.

The Na^+^ PMF profile in WT VSD (Figure 9, right, green line) shows a main high energy barrier (∼15 kcal/mol), and it is almost symmetrical with respect to it. This value is in line with results from extended umbrella sampling simulations of Na^+^ passage through the VSD of NavAb,^50^ where a single peak of ∼18 kcal/mol was obtained. In WT hKv7.2 VSD, the maximum is located in correspondence with the HCS (surrounded by residues V103, F137, V175) and hence well correlates with the lack of hydration of this region.

In the R207Q mutant, the PMF profile (Figure 9, right, magenta line) shows two maxima with an intermediate well, implying that the consequences of the substitution spread over the VSD structure. The main peak is in the same position as in the WT and reaches ∼8 kcal/mol, indicating that the mutation substantially lowers the main free energy barrier to ion passage. The interposed minimum is located between the position of the Cα atoms of residues E130 and V103, remarkably close to Q204, the glutamine that naturally replaces the arginine in position R3 in Kv7 VSDs.

**Figure 10:**
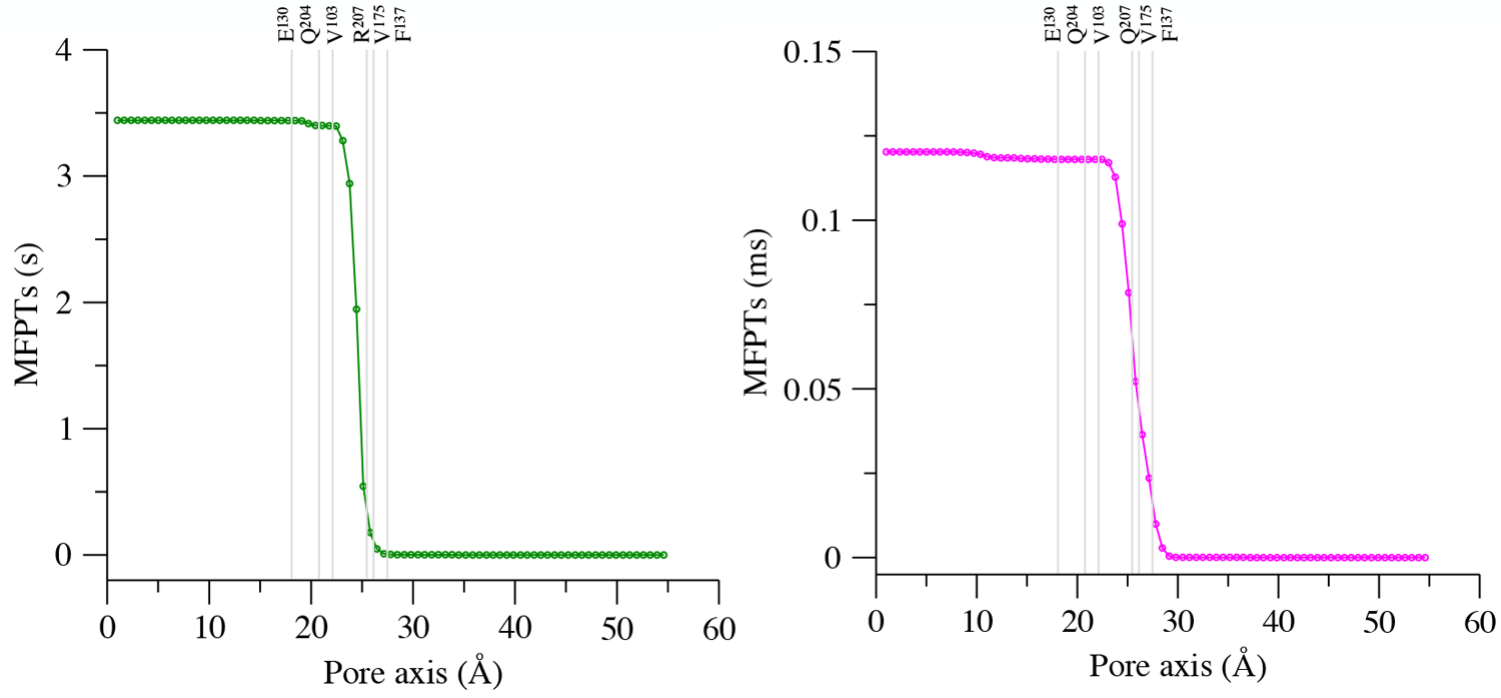
MFPTs from all milestones to the most intracellular one in the WT (left) and the R207Q VSD (right). The pore axis is oriented from the extracellular space to the intracellular one, and the position of the Cα atoms of relevant residues are represented as gray lines.

From the same VTMM simulations, we also obtained the MFPTs for Na^+^ transit from all milestones (Figure 10), starting with the most extracellular one, to the last one at the intracellular side, thus representing an inward ionic flux. As expected, the time scale of cationic permeation is dramatically different in the two systems, with the transition requiring the second time scale for the WT (3.4 s) and the millisecond time scale for the mutated VSD (0.12 ms), while in both cases a critical role is played by the HCS residues.

### Sodium Coordination during Permeation

We then analyzed the VTMM trajectories to investigate the coordination of the Na^+^ ion along the permeation pathway.

**Figure 11:**
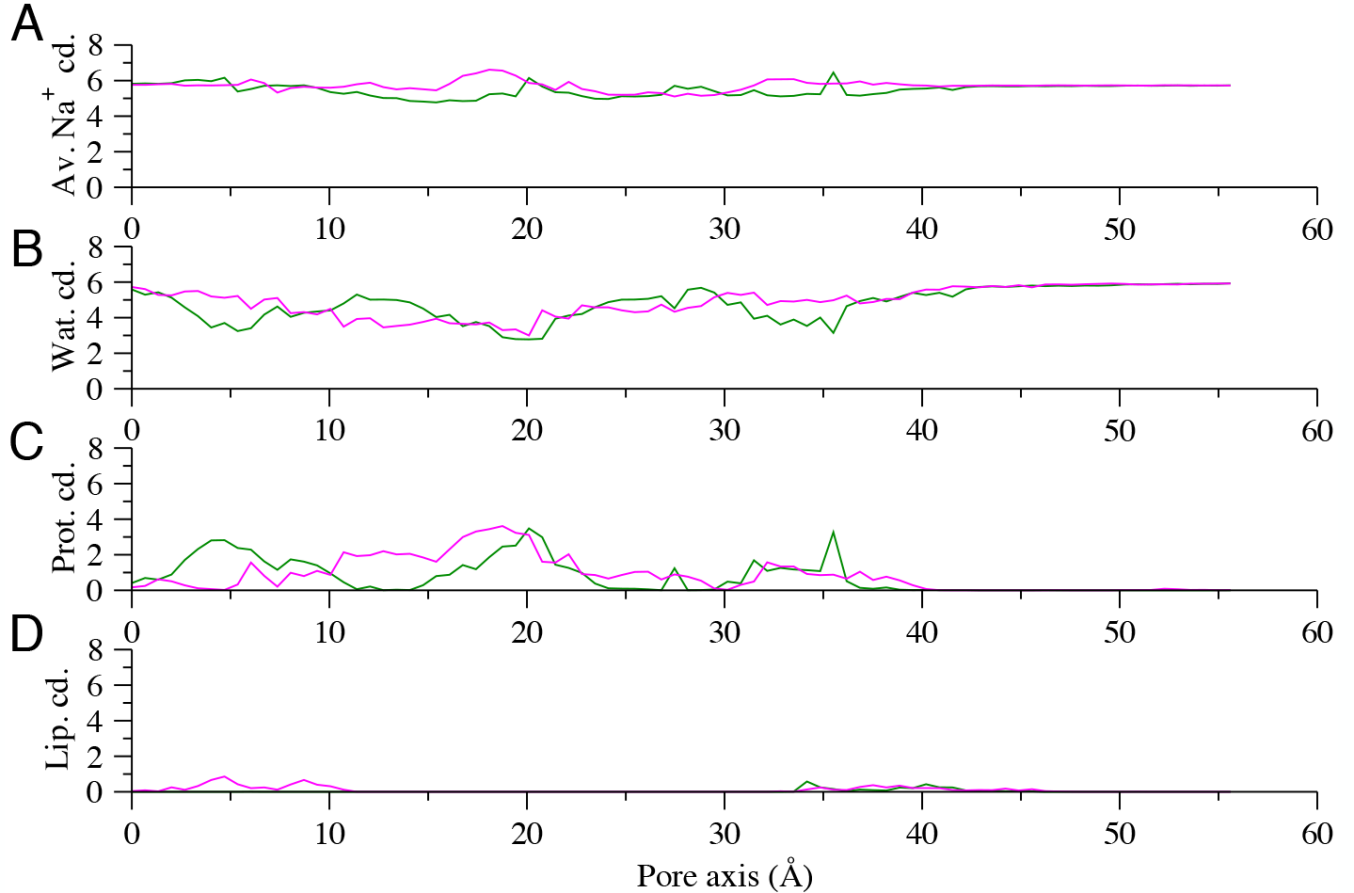
(A) Average total coordination of the Na^+^ ion as a function of the *z* direction parallel to the VSD axis for the WT (green curve) and the R207Q mutant (magenta). Different contributions to coordination are shown separately for water (B), protein atoms (C), and lipids (D).

The average total coordination number, as well as contributions from water, protein, and lipid atoms are reported in Figure 11. Results show a pattern of alternating coordination from water and protein atoms, resulting in a slightly reduced coordination in the HCS (*z* ∈ [20, 30] Å) in both systems.

At the extracellular side (*z* ∈ [0, 10] Å), the largest contribution to the Na^+^ solvation shell is provided by water molecules (Figure 11B) and, for the WT only, by additional interactions with the VSD extracellular loops (Figure 11C). However, these differences do not affect the PMF profile in this zone, where the two curves are comparable. Conversely, in the region defined by the interval *z* ∈ [10, 20] Å, characterized by marked differences between the two PMF profiles, we observe an increase of coordination from protein atoms in the mutant (Figure 11C). This correlates with the free energy minimum observed in this segment of the protein cavity, where the R207Q mutation allows the formation of more favorable interactions of the permeating ion with protein residues such as E130.

## Discussion

In the recent years, many studies have been devoted to clarify the structural and functional features of ω-currents.^25−27^ These leak currents are created by mutations affecting different voltage-gated ion channels and lead to a wide spectrum of channelopathies.^27^ In this context, various approaches based on MD simulations contributed to the understanding of the molecular effects of VSD mutations associated with ω-pores.^34,47,50^ All these efforts highlight the charge (cationic) selectivity and the dependence on the position of the R residues in the VSD S4 helix. However, these investigations were limited to a few representative systems.

In the case of the Kv7 channels family, it has been supposed that a single mutation affecting the arginine 207 (R4Q) is responsible for the destabilization of the VSD active configuration and the formation of a continuous hydrated pathway.^41^ Based on macroscopic and gating current measurements, it has been suggested that an inward Na^+^ current could be the final effect of the mutation, creating an abnormal depolarizing force that facilitates cellular hyperexcitability. However, a similar effect could also be generated by a flux of H^+^, rather than of a larger alkali. Indeed, the cavity of the VSD could not allow the passage of cations and prefer a proton transport between water molecules, via the so-called Grotthuss hopping mechanism. Therefore, detailed investigations are required to explain the subtle details of the conduction mechanism in the R207Q mutant.

We generated a high-quality model of the hKv7.2 VSD, since no experimental structures were available for the channel. To this aim, we employed a combined use of structural modeling approaches to generate a comprehensive set of refined models of the hKv7.2 VSD. The most promising candidate, based on the cryo-EM structure of the *Xenopus laevis* Kv7.1 channel^53^ (xKv7.1, PDB ID 5VMS) as template, was selected for all the MD simulations presented in this work. The structure was simulated in explicit solvent and in two different membrane system to monitor the effect of the environment on the structural features associated with an activated Kv7 VSD. Along the MD runs in both membrane systems, the model is characterized by reasonable RMSDs and stable interhelix cross-distances. Specifically, we observed a steady network of interhelix SBs anchoring both the extracellular- and the intracellular-facing regions of the activated VSD conformation, preventing hydration in the central HCS and forming a barrier to the passage of ions, as expected for the WT Kv7.2.

While we were finishing writing this work, a cryo-EM structure of hKv7.2 with the VSD in the activated state was published^54^ (PDB code 7CR0, 3.1 Å  resolution map, atomic model built *de novo*). A comparison between our model and the experimental structure is provided in the Supporting Information (Figure S14). Although the new experimental structure misses the extracellular S3–S4 linker, our VSD is nicely superimposable on it at the level of the transmembrane S1 to S4 helices. The side chains of the S4 arginines and of the facing acidic residues are all in closely similar positions, forming the predicted active state-stabilizing salt bridges. In particular, in the cryo-EM structure, R207NH1 and E130OE2 are at 3.4 Å, establishing the salt bridge we monitor here with the distance named *d*1. A difference is observed for the side chain of R210 (R5, Figure S14A,C), which in our model points toward E140 to form a stable SB but in the cryo-EM VSD adopts a different rotameric state, with its guanidinium group closer to the tip of the phenyl ring of F137, more sideways to the charge transfer center. It is important to note that, in most Kv active-state VSD structures, the positively charged residue at that position interacts with one of the two basic residues of the gating charge transfer center,^72,106^ formed in hKv7.2 by the residues F137, E140, and D172. Such interaction is also observed in the just published cryo-EM structure of the open hKv7.4 channel^107^ (overall resolution 2.5 Å, atomic model built using the human Kv7.1 coordinates to fit the map). Moreover, previous experimental investigations and structural modeling of Kv7.2 confirmed the occurrence of a direct interaction between R210 and E140, as in our model and described its loss as the structural determinant of the channel dysfunction observed in the E140Q mutant, the genetic cause of neonatal onset DEE.^24^ To better investigate any difference between our model and the experimentally obtained structure, we performed a 200 ns long unrestrained MD simulation of the hKv7.2 cryo-EM VSD embedded in the heterogeneous membrane. The RMSD of the backbone atoms of TM helices between the model and the experimental structure after the simulation is 1.3 Å, revealing the remarkable quality of our model. Interestingly, along the simulation of the cryo-EM VSD, R210 fluctuates between the conformation observed in the starting structure and one where it interacts with E140, as in our model (Figure S14D), with the distance *d*3 (between R210CZ and E140CD) being less than 5 Å for about 40% of the simulated trajectory. Although the configuration assumed by R210 in the cryo-EM structure is rarely found in other VSDs, it still occupies the space inside the sensor, hindering the access of water, a necessary condition for VSD functioning. Hence, even if the cryo-EM R210 conformation might induce local differences with our predicted structure, we believe these should not affect our main conclusions. We investigated the effects on Kv7.2 VSD conformation of a set of specific point mutations of clinical relevance. When R207 is mutated into a glutamine, we observed enhanced fluctuations in the RMSD profile, which as revealed by cross-distance analysis, are mainly associated with changes in the mutual arrangement of helices. Such changes induce an expansion of the HCS that facilitates the access of water molecules into the internal cavity, with the formation of a solvent wire across the transmembrane domain. These results are in agreement with those obtained from previously published, shorter MD simulations,^41^ thus confirming the observation over a longer time scale. We then studied a set of mutations affecting charged residues located in the portion of the protein facing toward the cytosolic space (R213W, R213Q, D212G, and E140Q). Previous experimental data^24,41^ demonstrated that mutations affecting the R213, D212, and E140 residues altered channel function and were associated with a decrease in stability of the active VSD conformation. Moreover, while involving S4 residues, the first three substitutions did not cause activation of leak currents.

It is then of relevance to explore the differences in the perturbation of the structure induced by the various substitutions. When applied to our model, these mutations strained the structure, as evidenced by increased fluctuations in the RMSD profiles, and augmented water occupancy in the HCS. However, the SB between the R207 and E130 side chains remains stable in all the simulations, avoiding the formation of a continuous water wire, contrary to what was observed in the R207Q system. To study the effect of the R207Q mutation and the possible connection with the formation of a transmembrane Na^+^ flux, we used the VTMM method to determine the thermodynamic and kinetic properties of cationic flux through the VSD, in the WT and the mutant protein. Although during the permeation process we observe only minor differences in sodium coordination by water in the two systems (Figure 11B), the WT PMF profile shows a high energy barrier (∼15 kcal/mol) near the HCS, consistent with the absence of ω-currents. A similar, even higher value (∼18 kcal/mol) was obtained using umbrella sampling for Na^+^ permeation through the WT VSD of NavAb.^50^ Also in that case the authors observed a marked local increase in water hydration in the WT VSD cavity during the restrained simulation, which did not imply however the formation of a pore and hence the possibility of leak currents. Such a high value of energy for sodium permeation in WT VSDs is the result of the combined effect of the presence of (i) a large number of positively charged residues in the S4 helix inducing electrostatic repulsion, (ii) the SB interaction between R207Q and E130, forming an obstruction in the VSD cavity that prevents the passage of water from the extracellular to the intracellular space, and (iii) the narrow hydrophobic fenestration in the HCS, which is welded at the top by the aforementioned SB, separating the two vestibules of the VSD and avoiding the formation of a water pore. Notably, all these structural features are modified in the R207Q system, where the mutation reduces the number of positively charged residues and breaks the SB, destabilizing the VSD conformation and inducing an expansion of the internal cavity allowing the water to access the cavity. From a thermodynamic point of view, the final effect is a net reduction of ∼8 kcal/mol in the energy peak. The PMF profile reveals an intermediate minimum in the VSD upper vestibule, located at the level of the Cα atoms of E130 and Q204 (the glutamine that naturally replaces the arginine in position R3 in the Kv7 VSDs), and hence related to an interaction of the cation with the now unpaired acidic residue. This feature points to a cooperative effect of the two S4 glutamines in the mutant, leading to a marked modification of its thermodynamic and kinetic properties of ion permeation. The existence of an intermediate minimum may raise the question as to whether it would result in a kinetic trap for the ion flux. In this respect, the calculation of MFPTs revealed the reduction of Na^+^ permeation time scale by 4 orders of magnitude in the mutant structure with respect to the WT. Given that our calculations are performed at zero transmembrane potential, this result opens the possibility of ω-currents emerging in the R4Q mutant at smaller (albeit still depolarizing) voltage values than in the WT channel, as obtained from electrophysiological measurements.^41^

Additional monitoring of Na^+^ ion coordination along the VTMM simulations allows linking of the PMF features with local interactions of the cation, revealing, in the mutant, the role of E130 in the region of the intermediate energy minimum. Interestingly, the interaction between E130 and sodium is favored by the concomitant absence of the positive charge R3, typical in the Kv7 structures bearing a glutamine (Kv7.2 Q204), since, when R207 is neutralized, no other basic partner is available nearby for E130, leaving it unpaired. Our data hence yield a description of the combined effect of the two glutamines in the S4 helix of the mutant that was postulated in the first work describing the formation of the ω-current in this system.^41^ There, the authors supposed that the absence of the R204 residue could facilitate the formation of the ω-pore, in agreement with experimental results demonstrating that the neutralization of two sequential S4 charges resulted in measurable ω-currents in Shaker channels^108^ and in a Nav1.2 VSD.^109^

## Conclusions

In this study, we used a combination of molecular modeling and MD simulations to reconstruct the structural features of a mutated Kv7.2 VSD associated with the formation of ω-currents at depolarized potentials (R207Q). We provide a refined structural model of Kv7.2 VSD that summarizes all the features of an activated configuration at depolarized potentials and that we employed to study the impact of a set of mutations. Extensive MD simulations of these mutants show that while some substitutions destabilize the active configuration of the VSD, only R207Q is associated with the formation a continuous water wire across the transmembrane domain, an essential condition for the formation of an ω-current. The ionic origin and selectivity of this leak flux are still not completely understood. Interestingly, in ref (110), it was pointed out that the combination of Q and E/D residues is a pivotal element of nonselective cation filters, and a similar situation could occur in the R207Q Kv7.2 mutant with the conserved Q204 and the unpaired E130. However, an inward sodium leak current remains a highly plausible origin of the persistent depolarization associated with the axonal hyperexcitability characterizing the R207Q mutant.^41^

In this context, our VTMM simulations provide a clear picture of the mechanistic events associated with the formation of the Na^+^ leakage in the pathological mutant. Moreover, our results demonstrate the ability of MD simulations to describe the molecular features related to the formation of ω-current in channelopathies, in line with results obtained for other systems. This *consensus* may underpin the profitable use of MD simulations to predict the effects of yet unidentified mutations. Furthermore, together with our findings on the ionic species involved in R207Q-induced ω-currents, we provide a set of reliable Kv7.2 structures, refined via extensive MD simulations, that complement the latest experimental achievements and may contribute to the understanding of Kv7.2 function under physiological and pathological conditions.

## Supporting Information Available

Stages of homology modeling of the WT hKv7.2 VSD from various templates and identification of the optimal model, standard MD simulations of the WT system in the HOM membrane (RMSD values, water occupancy, salt-bridge and cross distances analysis), MD simulations of the mutants R213W, R213Q, D212G, and E140Q (RMSD values, water occupancy, and cross distances analysis), details of TAMD and SMD simulations, and comparison between the hKv7.2 VSD model and cryo-EM structures of hKv7.2, xKv7.1, and hKv7.1 (PDF)
