# Supplementary material for: Structural Mechanism of ω-Currents in a Mutated Kv7.2 Voltage Sensor Domain from Molecular Dynamics Simulations
Source: J Chem Inf Model. 2021 Feb 11;61(3):1354–67. doi: 10.1021/acs.jcim.0c01407 (PMC8023575; doi:10.1021/acs.jcim.0c01407)
Supplement: Supplementary file 1 — ci0c01407_si_001.pdf [file ci0c01407_si_001.pdf]

Supporting Information for: Structural Mechanism of ω-Currents in a Mutated Kv7.2 Voltage Sensor Domain from Molecular Dynamics Simulations

Giulio Alberini,[†,‡] Fabio Benfenati,[*,†,¶] and Luca Maragliano[*,§,†]

†*Center for Synaptic Neuroscience and Technology (NSYN@UniGe), Istituto Italiano di Tecnologia, Largo Rosanna Benzi, 10, 16132, Genova, Italy*

‡*Department of Experimental Medicine, Università degli Studi di Genova, Viale Benedetto XV, 3, 16132, Genova, Italy*

¶*IRCCS Ospedale Policlinico San Martino, Largo Rosanna Benzi, 10, 16132, Genova, Italy*

§*Department of Life and Environmental Sciences, Polytechnic University of Marche, Via Brecce Bianche, 60131, Ancona, Italy*

E-mail: fabio.benfenati@iit.it; l.maragliano@univpm.it

Methods and results - Molecular modeling and standard MD simulations of the WT human Kv7.2 system

Choice of the templates. We built and tested five distinct homology models of the human Kv7.2 (hKv7.2) VSD (hereafter named M1 to M5), using four different templates as described in the following:

1. The first template is the depolarized state of the mammalian Kv1.2 VSD that was previously equilibrated and refined using MD simulation with explicit solvent and lipid membrane.[1] The atomic structure of this VSD is obtained from the open/active channel structure that was first generated by Pathak et al.[2] from the X-ray coordinates of the rat Kv1.2 (rKv1.2) (PDB ID: 2A79[3]), after completing missing structural information. This structure, which has a low sequence identity of $\sim$ 22 % with Kv7 channels, was used to build two hKv7.2 VSD models with different homology algorithms (denoted in the following as M1 and M2, respectively).

2. The second template is a refined model of an activated VSD from a human Kv7.1 (hKv7.1) channel published by Smith et al.,[4] obtained using as template the refined version of the rKv1.2 structure described above. The sequence identity between hKv7.1 and hKv7.2 is 60 %, and the hKv7.1 model was already used as a template to build a Kv7.2 tetramer in Ref. 5. We named M3 our model obtained from the hKv7.1 template.

3. The third template comes from the chimeric K^+ channel in which the voltage-sensor paddle (corresponding to the S3b-S4 region) of the Kv2.1 VSD was transferred into the Kv1.2 subunit (Kv1.2/Kv2.1 chimera, PDB ID: 2R9R,[6] with a 2.4 Å resolution) to overcome the limitations of the PDB 2A79 structure, thus obtaining a full experimental structure of an open channel configuration with activated VSDs. The Kv1.2/Kv2.1 VSD was used to model Kv7 structures in various other works.[7–9] The chimeric structure also has low sequence identity with Kv7 channels. We used the chimera to design

the hKv7.2 VSD model M4.

4. The fourth template is the recently solved cryo-EM structure of the *Xenopus laevis* Kv7.1 channel (xKv7.1, PDB ID: 5VMS,[10] 3.7 Å resolution). The structure corresponds to the so-called decoupled state at depolarized potential, with a closed pore and the VSDs in an activated conformation. The HHPRED webserver[11,12] identified this structure as the most promising candidate for modeling a Kv7.2 channel, thanks to its high sequence similarity with hKv7.2. Because of its incomplete structure and low resolution, before using it as a template we refined the xKv7.1 conformation by modeling *ab initio* the missing residues in the S3-S4 extracellular loop via the RCD+ webserver.[13,14] We then used the refined structure to design the hKv7.2 VSD model M5. Recently, a cryo-EM structure of the human Kv7.1 with activated VSDs was published, with PDB ID: 6UZZ.[15] Since the structural differences between the VSDs of xKv7.1 and of the new hKv7.1 (residues 109 to 235) are quite small (the backbone RMSD is about 1 Å), we believe using this new hKv7.1 as a template should not change significantly our model M5.

Sequence alignments and preparation of the Kv7.2 models. Sequence alignments were used to identify protein regions of similarity and guide the structural modeling. A multiple sequence alignment between Kv7 sequences and the templates was generated with Clustal Omega[16] and pairwise sequence alignments between the templates and the targets were generated using the SWISS server.[17]

SWISS-MODEL[17] pipelines, based on a rigid fragment assembly approach, were used to build all the models, except M2 that was designed with MODELLER.[18,19] Unlike SWISS-MODEL, MODELLER implements a method, named *satisfaction of spatial restraints*, by which a set of geometrical criteria are used to guide the location of each atom of the model. The structures of the five models were refined with FG-MD[20] producing five final configurations which we indicate as S_0^{M1-M5} (Structure at $t = 0$ for models 1 to 5). FG-MD is

a molecular dynamics (MD)-based algorithm to refine atomic-level models of protein structures by identifying analogous fragments from the PDB and improve the local geometry, the torsion angles and the hydrogen-binding networks.

System setup for preliminary MD simulations and selection of the representative model. After modeling, all the hKv7.2 VSD structures were further refined using MD simulations. Each model was embedded in an explicit heterogeneous membrane and solvated by explicit water molecules using the CHARMM-GUI server.[21,22] The MD ensemble, algorithms and parameters were those described in Main text for all simulations.

Each system was equilibrated using a multi-step equilibration protocol. First, the CHARMM-GUI equilibration procedure was performed, which consists in performing runs using restraints to gradually release the system to facilitate stable simulation. Then, the system was equilibrated for additional 30 ns (10 ns with protein heavy atom restraints, 10 ns with protein backbone restraints, and 10 ns with Cα restraints only). Finally, for each model, we performed an unrestrained MD run of 10 ns.
We checked for the presence of the following VSD stabilizing salt bridges:[6–10,23,24]

- R207$^{\mathrm{S4}}$ - E130$^{\mathrm{S2}}$;
- R210$^{\mathrm{S4}}$ - E140$^{\mathrm{S2}}$;
- R213$^{\mathrm{S4}}$ - D172$^{\mathrm{S3}}$;
- R210$^{\mathrm{S4}}$ - D172$^{\mathrm{S3}}$;

where the superscript indicates the helix in which residue is located.

The structures M1 to M3 were excluded from the pool of models because they do not include, in the starting S_0^{M1-M3} configuration and/or during the entire test simulation, one or more of the last three salt bridges.

To choose between the M4 and M5 structures we evaluated the persistence of the first salt

bridge of the list, between residues R207 (R4) and E130. This interaction is an important feature of the active VSD configuration at depolarized potentials, and it is responsible for the focused electric field in the membrane environment. We thus calculated along the short unrestrained MD simulations of M4 and M5 the values of the distance (named d1) between the most external carbon atoms of the interacting side chains, *i.e.* R207CZ^{S4} and E130CD^{S2}, and we observed larger fluctuations in M4. In particular, we obtained average d1 values of 5.65 ± 0.90 Å and 4.00 ± 0.10 Å, for M4 and M5, respectively. Based on this analysis, we finally chose M5 as our best hKv7.2 VSD model, and employed it in all the MD simulations described in this work.

Methods and results - MD simulations of the WT model

A representative snapshot of the optimal system in its membrane environment is shown in **Figure S1**.

As described in Main text, we generated an heterogeneous (HET) and an homogeneous (HOM) membrane system. To exclude differences in the leaflet/water interfaces induced by the different membrane composition, we calculated the number of water molecules within 10 Å from each lipid layer or from the protein during the unrestrained MD simulations in the HET and HOM systems. Results are shown in **Figure S2** and confirm that there are no appreciable differences between the two water layers in the HET system, and that the extent of the fluctuations do not differ from what observed in the homogeneous membrane setup.

To check the structural stability of our model in both membrane environments, as well as its reliability as a representative of the active VSD state, we calculated root mean squared deviation (RMSD) values, distances between facing, interacting side chains, cross distances between backbones of residues from different TM helices, and water occupancy inside the VSD. Results are reported in the following.

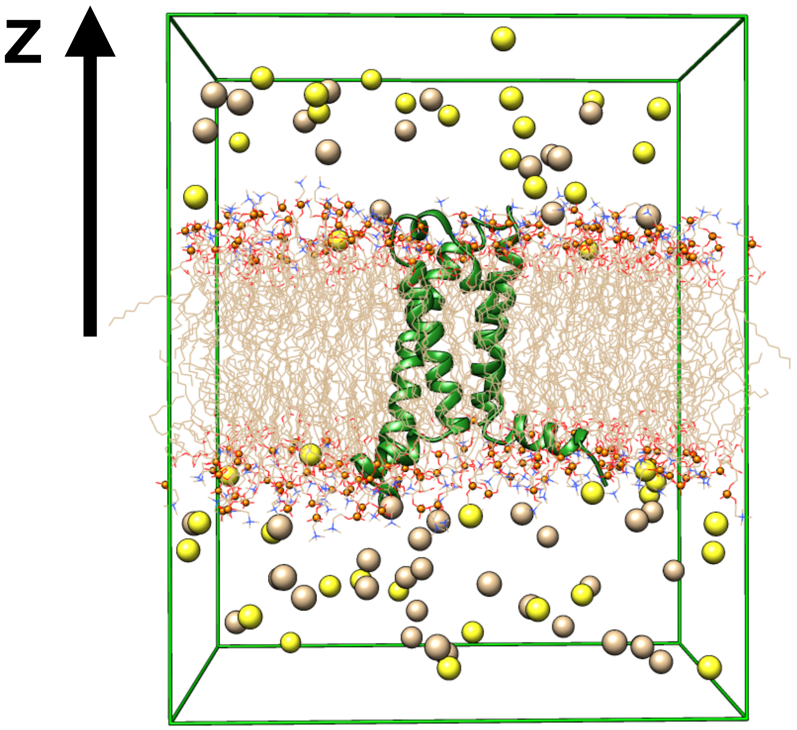

Figure S1: Snapshot of the equilibrated VSD domain (green ribbons) in the cell. Explicit membrane is introduced using brown wires and orange phosphorus atoms. Ions are shown as yellow (sodium) and grey (chloride) spheres. Water molecules are not included for clarity. The coordinate z is orthogonal to the membrane plane.

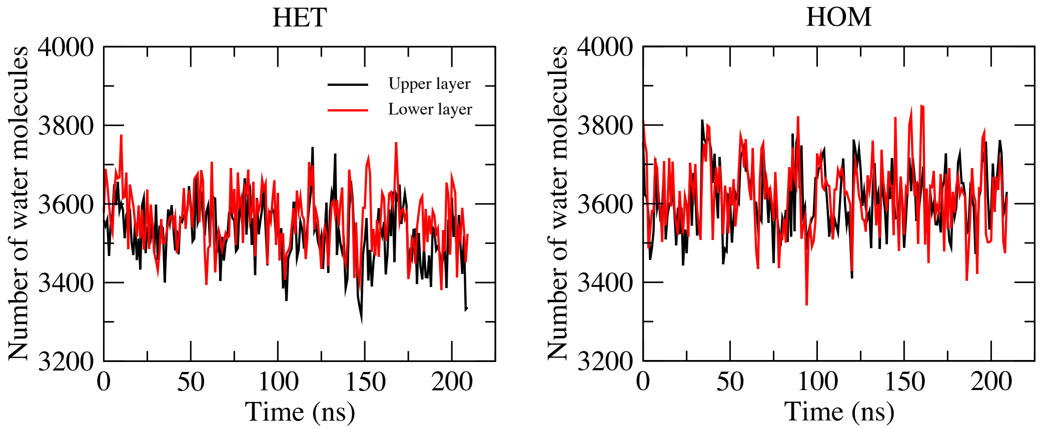

Figure S2: Number of water molecules within 10 Å from each lipid layer and the protein in the unrestrained MD simulations in the HET (left) and HOM (right) systems.

RMSD analysis. RMSD values of the VSD helices backbone atoms with respect to the starting S_0^{M5} configuration were calculated along the unrestrained, 210 ns-long trajectories,

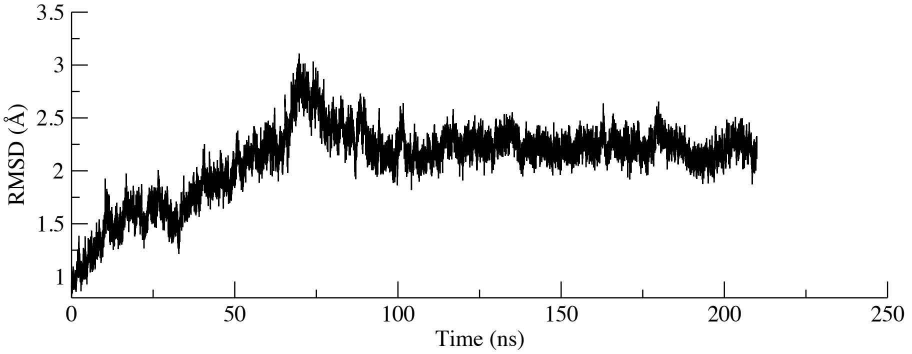

Figure S3: Backbone RMSD values of the TM α-helices for the HOM system.

and are reported in **Figure S3**. They show a plateau at ~ 2.25 Å revealing a stable VSD structure.

Salt bridges analysis. We monitored the persistence in time of a set of interactions, including the salt bridges (SBs) known to characterize the Kv7.2 VSD state at depolarized potentials.[6–10,23,24]

As a measure, we used the distance between the most external carbon atoms on the side chains of interacting residues. In Kv7 channels, a glutamine (Q204 in Kv7.2) is found at the position corresponding to one of the SB-forming S4 arginine residues of other Kv channels. For this reason, we included in the list also the distance between Q204 and E130, even if they do not form a SB:

- R207CZ^{S4} - E130CD^{S2}, named d1;
- Q204CD^{S4} - E130CD^{S2}, named d2;
- R210CZ^{S4} - E140CD^{S2}, named d3;
- R213CZ^{S4} - D172CG^{S3}, named d4.

When the VSD is in the active conformation, the residues involved in d1 and d2 are located in the upper water vestibule, while all the other residues are located in the lower vestibule. The presence of the SBs described by d1, d3 and d4 is assumed to characterize the active Kv7.2

VSD state, and in particular the SB R207CZ^{S4} - E130CD^{S2} (described by d1) is considered pivotal for the stability of the VSD active conformation.[23] The described distances were calculated along the unrestrained trajectories of both HOM and HET WT systems, using frames extracted every 10 ps. The time evolution of the most relevant d1-d4 distances is shown in the Main text and in **Figure S4** for HET and HOM systems, respectively, and the corresponding mean values are reported in **Table S1**. This analysis reveals a stable active VSD structure along the unrestrained trajectories, with minor differences between the two membrane environments that do not affect the most important interactions (in particular those represented by d1, d3 and d4).

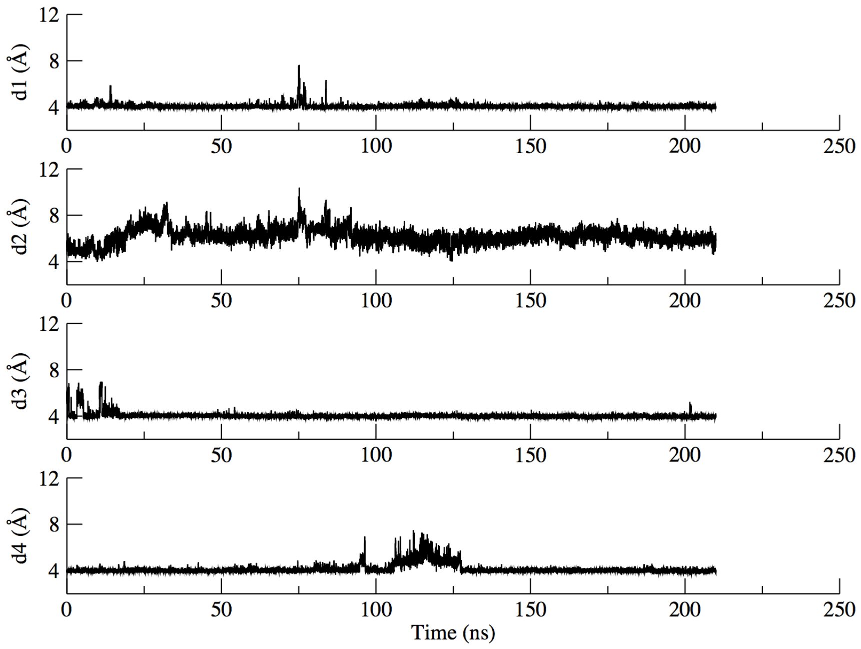

Figure S4: Time evolution of the d1-d4 for the HOM system.

Table S1: Analysis of the d1-d4 distances during the unrestrained simulations. For each distance we report the starting values measured for the S_0^{M5} structure and the average ± the standard deviation along the HET and the HOM MD runs. The distances are also calculated for the recent human Kv7.2 structure[25] (PDB ID: 7CR0).

System	d1 (Å)	d2 (Å)	d3 (Å)	d4 (Å)
S_0^{M5}	4.00	4.73	4.33	4.04
HET	4.40 ± 0.39	4.99 ± 0.64	4.08 ± 0.20	4.15 ± 0.35
HOM	4.06 ± 0.18	6.17 ± 0.66	4.02 ± 0.27	4.12 ± 0.40
PDB ID: 7CR0	5.62	4.42	6.48	5.71

VSD hydration analysis. VSDs are characterized by a hydrophobic constriction site (HCS) at their core, that separates an upper and a lower hydrated vestibule. We monitored the water occupancy within the VSD by using two criteria of increasing precision.

First, we calculated the number of water molecules with axial coordinate within the interval -5 Å$< z - z_0 < 5$ Å, where z_0 is the z coordinate of the center of mass (COM) of the group of atoms formed by the backbone of residues V103 - F137 - V175 - R207. Since water molecules do not diffuse in the membrane, this number counts water molecules that are in the upper and lower water vestibule of the VSD, and an increase in its value would imply structural instability of the domain. Then, we also calculated the number of water molecules inside a restricted region around the HCS, defined as a sphere of radius 5 Å and centered at the COM of the group of atoms described above. In a stable, active VSD structure the number of water molecules in this region should be very small and stationary over time. The time-evolution of the water occupancy calculated in the two ways is shown in **Figure 4** (Main text) and **Figure S5** for the HET and HOM trajectories, respectively. In both simulations, the number of water molecules is always quite small and stationary, in particular when the restricted sphere is used (black lines in **Figure S5**), confirming again the structural stability of our model. In particular, the values of the water counting in the sphere fits with the results described for the hydration of the HCS in another WT VSD type (3.9 ± 0.8).[26]

Cross distances analysis. To further evaluate the stability of the VSD model structure, we monitored a set of cross distances, *i.e.* distances between Cα atoms of residues on facing helices. These distances are defined as:

- V99CA^{S1} - D172CA^{S3}, named cd1;
- V103CA^{S1} - V175CA^{S3}, named cd2;
- S110CA^{S1} - V182CA^{S3}, named cd3;
- Y141CA^{S2} - M211CA^{S4}, named cd4;

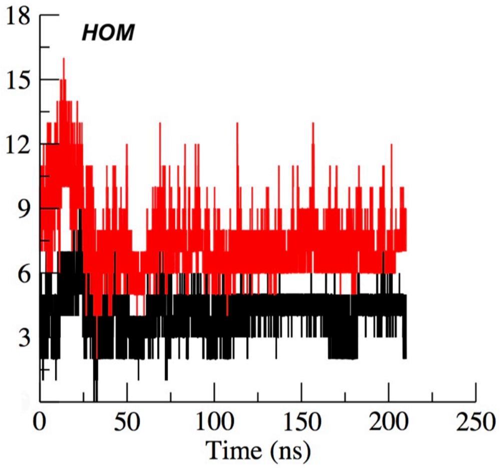

Figure S5: Water occupancy analysis for the MD simulations of the HOM WT VSD. Black curves describe the hydration of the HCS spherical core, while the red curve describes the hydration of the more extended interval (see text).

- I134CA^{S2} - L203CA^{S4}, named cd5;

The time-evolution of the cross distances is shown in main text and **Figure S6** for HET and HOM systems, respectively, and the corresponding mean values are reported in **Table S2**. The stationarity of these measures over time further confirms the stability of our model.

Table S2: Analysis of the CDs introduced in the text (cd1-cd5). The first row describes the cd value for the starting configuration of the model (S_0^{M5} configuration). The second and third columns show the average ± the standard deviation for these quantities during the last 190 ns of simulations for the system in the heterogenous (HET) and the homogeneous (HOM) membrane, respectively.

System	cd1	cd2	cd3	cd4	cd5
S_0^{M5}	13.80	12.58	14.91	13.12	10.55
HET	13.47 ± 0.46	13.23 ± 0.43	15.36 ± 0.70	14.20 ± 0.55	11.71 ± 0.56
HOM	13.19 ± 0.83	13.93 ± 0.80	15.69 ± 1.03	13.24 ± 0.76	12.56 ± 0.60

Methods and results - MD simulations of the additional mutants

We then introduced a set of additional Kv7.2 mutations affecting charged residues located in the more distal portion towards the cytosolic space (R213W, R213Q, D212G, and E140Q).

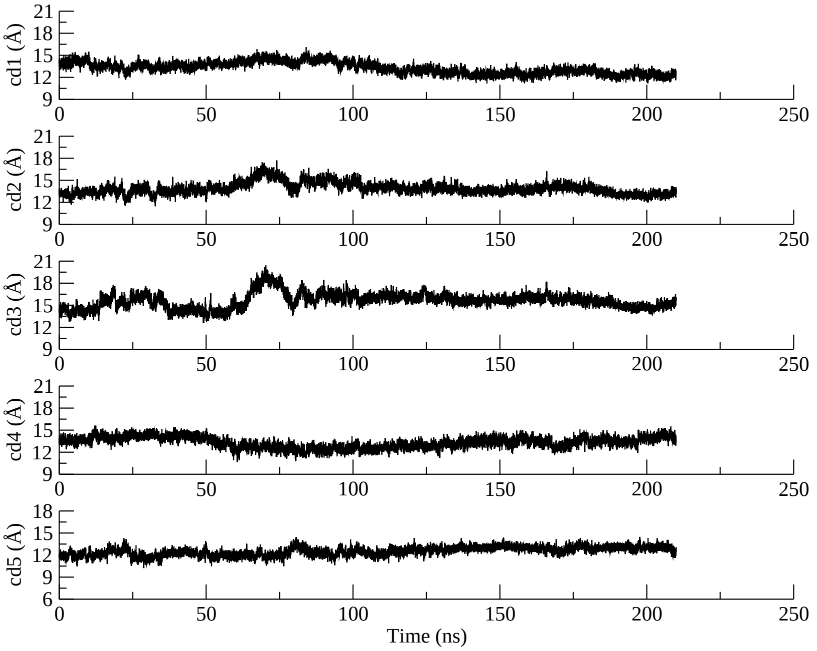

Figure S6: Cross distances in the HOM system.

All these mutants have clinical importance.[23,27–30] Previous experimental data[23] demonstrated that Kv mutations affecting the R6 and the D1 residues (R213W, R213Q and D212G in our Kv7.2 system) decrease the stability of the active voltage-sensing domain configuration, but do not generate an ω-current. In another recent work,[30] the E140Q Kv7.2 mutation was shown to be involved in the destabilization of the active VSD configuration.

System setup and RMSD calculations. All the systems were prepared for MD, following the procedure used for the R207Q model (heterogenous membrane). Before production, each system was further relaxed in the membrane environment with an additional 10 ns restrained equilibration. Finally, unrestrained 500 ns MD trajectories were produced for each system using the same MD setup of the unrestrained simulation of the WT system.

The RMSD versus time for the VSD backbone (TM α-helices) are reported in **Figure S7** for the four mutants. The E140Q system shows the greater structural instability during the unrestrained MD, without reaching a stable plateau in the time window considered.

VSD hydration and cross distance analysis. Water hydration was evaluated, following the

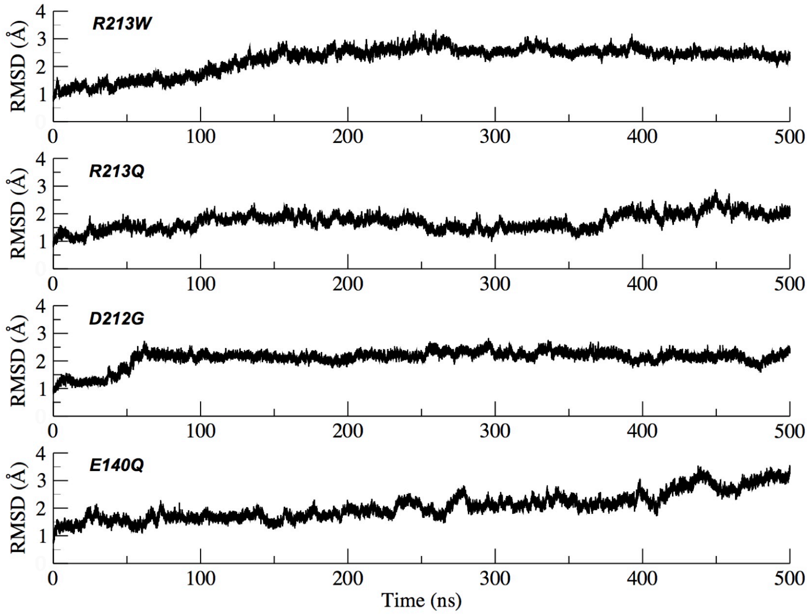

Figure S7: RMSD values for the VSD backbone of the TM α-helices for the four mutants affecting charged residues in the lower portion of the transmembrane domain.

same criteria introduced for the previous systems (**Figure S8**). In all mutants, we observe an increase of the hydration within the VSD with respect to the WT system, with more pronounced fluctuations for R213Q and E140Q. Analysis of the cross distances between the TM α-helices (**Figures S9 - S12**) demonstrates that, particularly for E140Q, the VSD cavity gets larger, and can host a higher number of water molecules. Since the mutations do not affect the SB described by the d1 distance, we monitored d1 along the trajectories (**Figure S13**), revealing a stable interaction between R207 (R4) and E130.

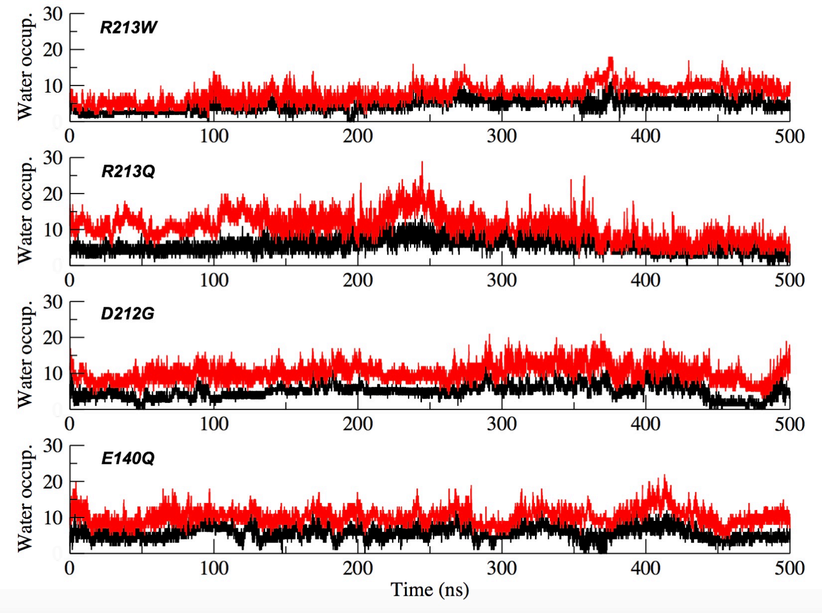

Figure S8: Water occupancy for the four mutants located in the more distal portion of the VSD towards the cytosolic space. Black curves describe the hydration for the HCS spherical core only, and the red ones the hydration of the whole HCS.

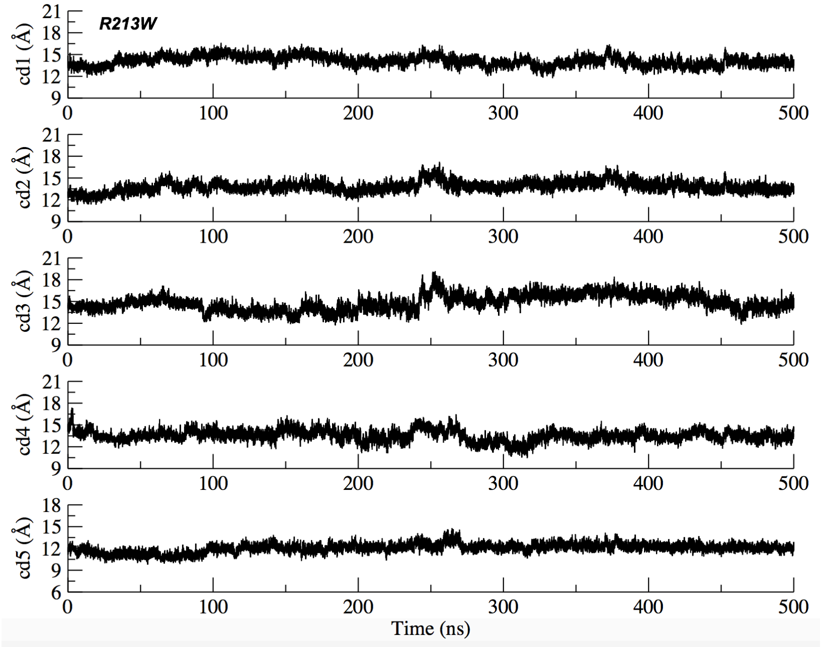

Figure S9: Cross distance interactions of the R213W model.

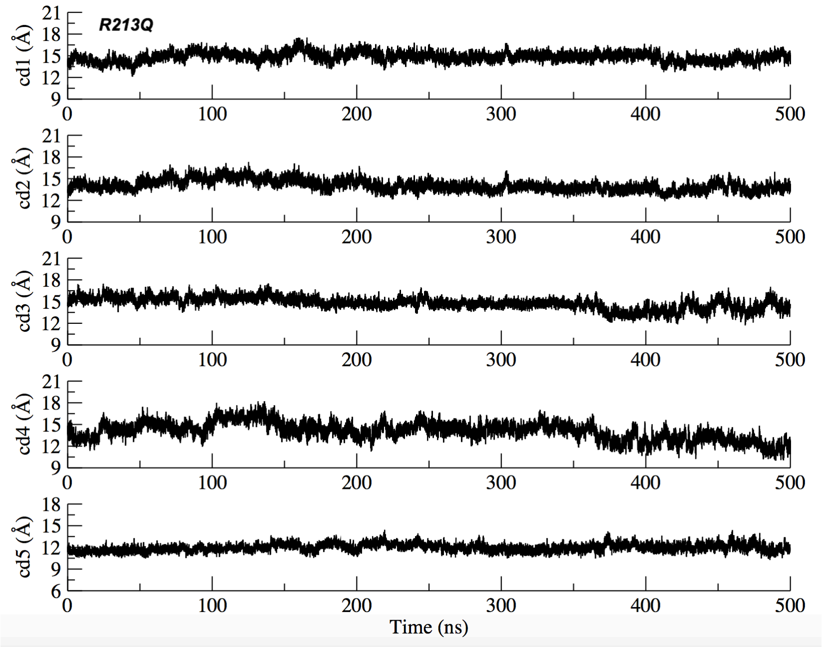

Figure S10: Cross distance interactions of the R213Q model.

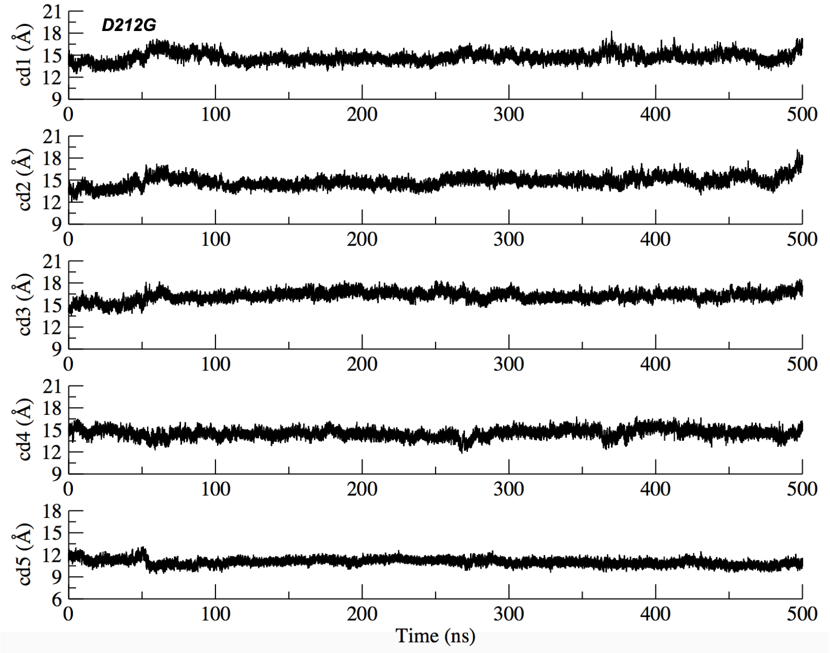

Figure S11: Cross distance interactions of the D212G model.

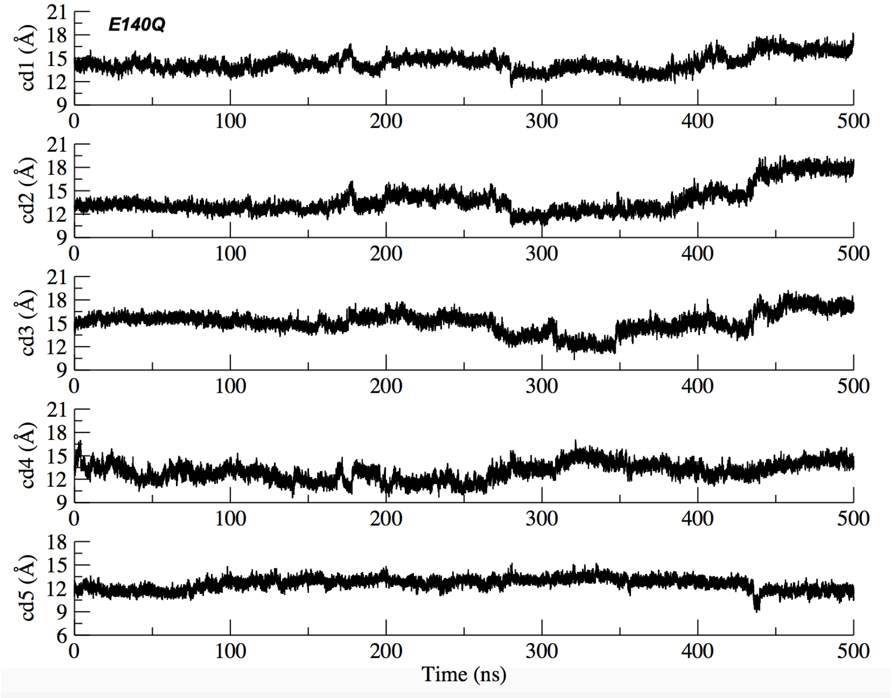

Figure S12: Cross distance interactions of the E140Q model.

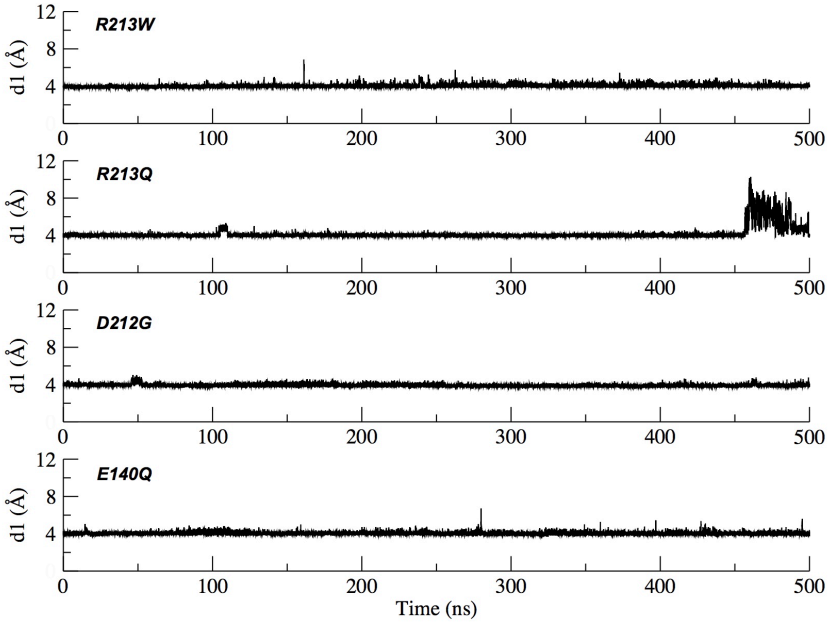

Figure S13: Time evolution of the d1 distance for the additional four mutants.

Methods for exploring the ionic pathway

Temperature Accelerated Molecular Dynamics (TAMD). In this work, we used TAMD[31] to generate multiple configurations of the Na^+ ion inside the mutated VSD cavity, as starting points for VTMM-MD simulations. For a system with n degrees of freedom, whose position in configuration space $\Omega \subseteq \mathbb{R}^n$ will be denoted by $\vec{r}$, we consider a set of N collective variables (CVs) $\vec{\theta}(\vec{r}) = (\theta_1(\vec{r}), ..., \theta_N(\vec{r}))$.

In a TAMD run, the system is extended to include $\vec{z}$ variables that also act as anchor points for CVs via a potential energy $U_\kappa(\vec{r}, \vec{z}) = V(\vec{r}) + \frac{1}{2}\kappa|\vec{\theta}(\vec{r}) - \vec{z}|^2$, where $V(\vec{r})$ is the CHARMM Force Field (FF) in our case, and $\kappa > 0$ is an adjustable spring-constant-like parameter. The extended system evolves according to the following dynamic equations:

$$\begin{cases} m\ddot{\vec{r}} = -\nabla V(\vec{r}) - \kappa \sum_{\alpha=1}^{N} (\theta_\alpha(\vec{r}) - z_\alpha)\nabla\theta_\alpha(\vec{r}) + \text{thermostat at } \beta^{-1} \\ \bar{\gamma}\dot{\vec{z}} = \kappa(\vec{\theta}(\vec{r}) - \vec{z}) + \sqrt{2\bar{\gamma}\bar{\beta}^{-1}}\vec{\eta}(t) \end{cases} \tag{1}$$

where m is the mass, $\vec{\eta}(t)$ is a Gaussian process with mean 0 and covariance $\langle \eta_\alpha(t)\eta_{\alpha'}(t')\rangle = \delta_{\alpha\alpha'}\delta(t - t')$, $\bar{\gamma}$ is a friction coefficient and $1/\bar{\beta}$ is an artificial temperature with $1/\bar{\beta} > 1/\beta$, where $1/\beta$ is the inverse of the temperature k_BT. As shown in Ref. 31, by choosing the parameter κ so that $\vec{z}(t) \sim \theta(\vec{r}(t))$ and the friction coefficient $\bar{\gamma}$ so that $\vec{z}$ moves slower than $\vec{r}$, one can generate a trajectory $\vec{z}(t)$ that evolves at the artificial temperature $1/\bar{\beta}$ on the PMF surface calculated at the physical $1/\beta$. By setting $1/\bar{\beta} > 1/\beta$, the $\vec{z}(t)$ trajectory will be able to cross PMF barriers unaccessible at the physical temperature and thus visit hidden metastable states.

Steered Molecular Dynamics (SMD). We also used SMD[32,33] to generate multiple configurations of the Na^+ ion inside the mutated VSD cavity, as starting points for VTMM-MD simulations. The cation is pulled towards the cytoplasmic side of the membrane at a constant velocity, using as CV the projection of the Na^+ ion on the VSD axis, z, normal to the

membrane plane. In SMD simulations the system is steered by applying an external force from an equilibrium state characterized by an initial CV value, $z = z_0$, to a new equilibrated state at $z = z_1$, following a non-equilibrium transition. The energy potential $U_{\check{\kappa}}(\vec{r}, z)$ includes, in addition to the physical energy $V(\vec{r})$ described by the CHARMM FF, a further harmonic forcing potential such that:

$$U_{\check{\kappa}}(\vec{r}, z) = V(\vec{r}) + \frac{\check{\kappa}}{2}(vt - (z(\vec{r}) - z_0))^2 \tag{2}$$

where v is the pulling speed.

Simulations setup. A series of TAMD and SMD simulations were used to induce the passage of a single Na^+ ion through the VSD cavity, starting from two snapshots of the WT and the R207Q mutant extracted from the standard MD simulations (heterogeneous membrane). To limit the lateral movements of the ion in the xy plane, an additional flat-bottom cylindrical restraint for the Na^+ ion was added.

Multiple TAMD simulations of different length were performed starting with the sodium ion in the extracellular space, close to the VSD. We used a time step of 2 fs, a fictitious friction $\bar{\gamma}$ of 50 ps^{-1} on the collective variable (the position of the ion along the pore axis), a fictitious thermal energy of $\bar{\beta}^{-1} = 7 - 10$ kcal/mol and a spring constant κ of 200 kcal/(mol $\cdot$ Å^2) (following the notation in Eq. 1). The user defined forces functionality in NAMD and the COLVAR module were used to perform TAMD simulations.

For SMD runs (Eq. 2) we chose $v = 10$ Å/ns and $\check{\kappa} = 5$ kcal/(mol $\cdot$ Å^2) and used Langevin dynamics and a time step of 1 fs. The SMD module of NAMD was used for these simulations.[34] To avoid any rigid body rotational or translational displacement of the protein, we used the same set of soft harmonic restraints on the Cα atoms introduce above already introduced for the production run.

Comparison between the WT Kv7.2 VSD model and cryo-EM structures

We compare here our model of the hKv7.2 VSD structure (active state) with the sensor from the recent cryo-EM hKv7.2 channel (PDB ID: 7CR0,[25] **Figure S14**), before and after unrestrained MD simulation of the latter. To run the simulation, the structure in the PDB file was first completed by modeling the missing S3-S4 extracellular loop via the RCD+ webserver.[13,14] The protein was then embedded into a heterogeneous lipid patch like the one used for the other systems and solvated with explicit TIP3P water molecules. The total charge was neutralized with a 150 mM NaCl solution. The CHARMM-GUI default equilibration procedure was performed, followed by 200 ns of unrestrained MD simulation in the NPT ensemble (same parameters of the previously described unrestrained simulations). The backbone RMSD of TM helices between our model and the simulated cryo-EM VSD calculated after optimal alignment, is 1.3 Å (residues 93 to 185 and 198 to 213 were considered, *i.e.* excluding only the reconstructed S3-S4 loop and the S4-S5 linker).

Finally, we show the comparison of our model with the sensor from *Xenopus laevis* Kv7.1 (our template, PDB ID: 5VMS,[10] **Figure S15**) and the human Kv7.1 (PDB ID: 6UZZ[15] **Figure S16**).

To facilitate the comparison between conserved residues in different amino-acid sequences, we will use here the general notation introduced in the Main text.

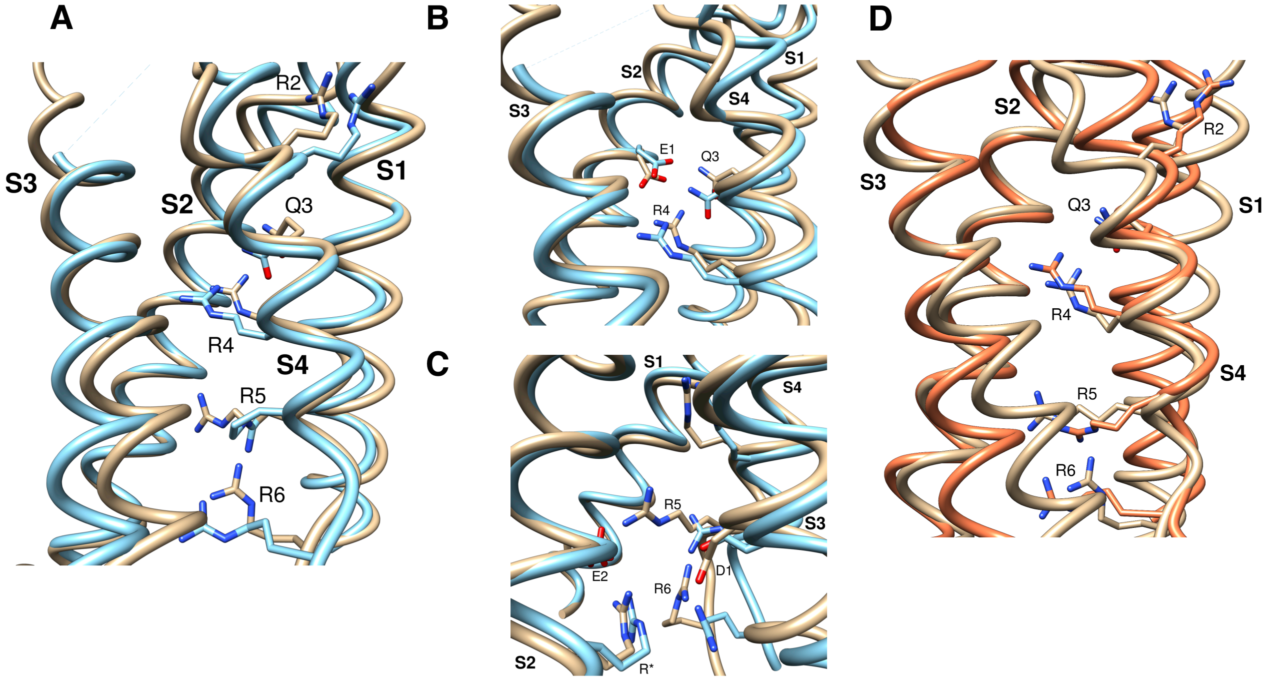

Figure S14: **A)** Superposition of our hKv7.2 VSD structural model (brown ribbons) and the VSD from the hKv7.2 cryo-EM structure (PDB ID: 7CR0, cyan). Side chains of arginine residues R2-R6 and glutamine Q3 are shown as sticks. **B-C)** Enlarged views illustrating the residues that define the d1-d4 distances in the upper and lower vestibule, respectively. **D)** Superposition of our model (brown) and the VSD from cryo-EM (orange) after unrestrained MD simulation.

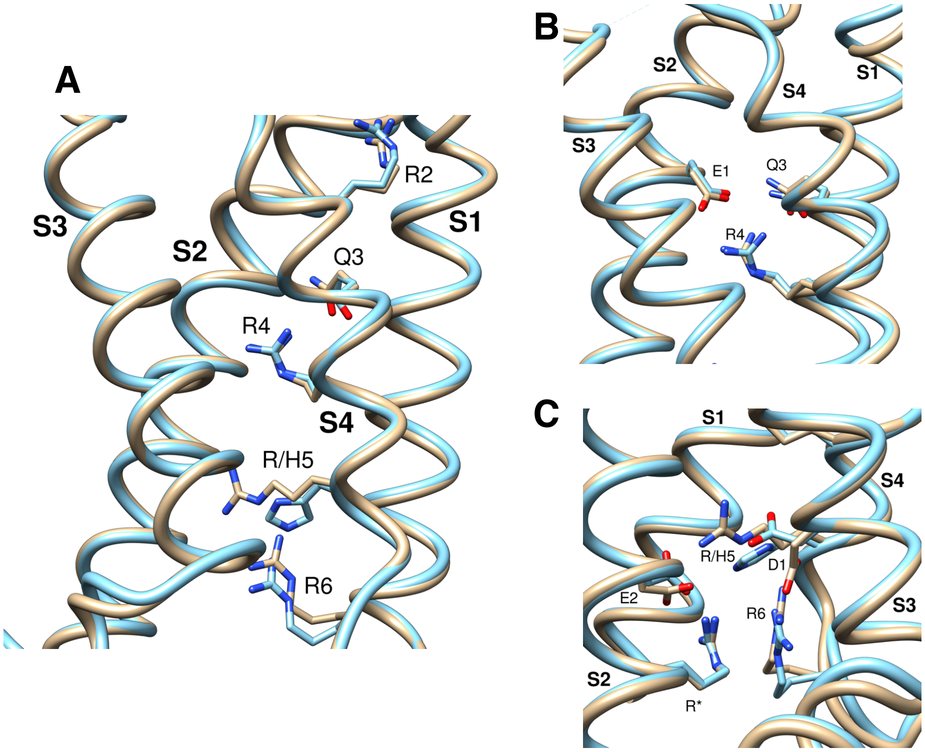

Figure S15: A) Superposition of our hKv7.2 VSD structural model (brown ribbons) and the VSD from the cryo-EM structure of the *Xenopus laevis* Kv7.1 channel (PDB ID: 5VMS, cyan). Side chains of arginine residues R2-R6 and glutamine Q3 are shown as sticks. **B-C)** Enlarged views illustrating the residues that define the d1-d4 distances in the upper and lower vestibule, respectively.

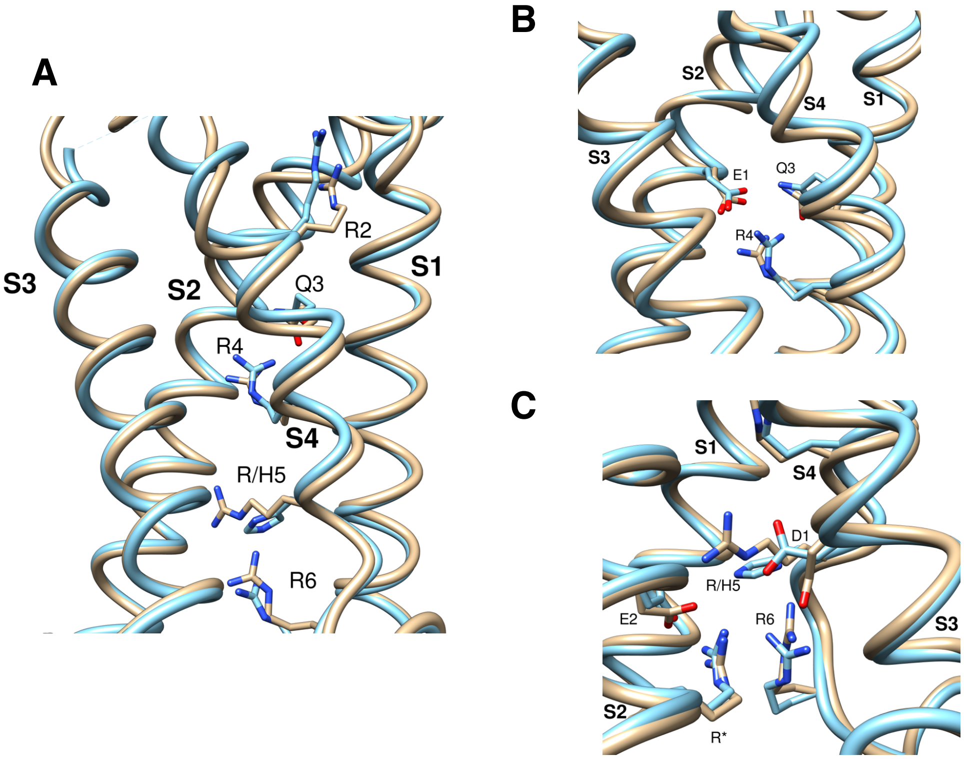

Figure S16: A) Superposition of our hKv7.2 VSD structural model (brown ribbons) and the VSD from the cryo-EM structure of hKv7.1 (PDB ID: 6UZZ, cyan). Side chains of arginines R2-R6 and glutamine Q3 are shown as sticks. **B-C)** Enlarged views illustrating the superposition between the residues that define the d1-d4 distances in the upper and lower vestibule, respectively.

References

(1) Khalili-Araghi, F.; Jogini, V.; Yarov-Yarovoy, V.; Tajkhorshid, E.; Roux, B.; Schulten, K. Calculation of the Gating Charge for the Kv1.2 Voltage-Activated Potassium Channel. *Biophys. J.* **2010**, *98*, 2189–2198.

(2) Pathak, M. M.; Yarov-Yarovoy, V.; Agarwal, G.; Roux, B.; Barth, P.; Kohout, S.; Tombola, F.; Isacoff, E. Y. Closing in on the Resting State of the Shaker K+ Channel. *Neuron.* **2007**, *56*, 124–140.

(3) Long, S. B.; Campbell, E. B.; MacKinnon, R. Crystal Structure of a Mammalian Voltage-Dependent Shaker Family K+ Channel. *Science.* **2005**, *309*, 897–903.

(4) Smith, J. A.; Vanoye, C. G.; George, A. L.; Meiler, J.; Sanders, C. R. Structural Models for the KCNQ1 Voltage-Gated Potassium Channel. *Biochemistry.* **2007**, *46*, 14141–14152.

(5) Peretz, A.; Pell, L.; Gofman, Y.; Haitin, Y.; Shamgar, L.; Patrich, E.; Kornilov, P.; Gourgy-Hacohen, O.; Ben-Tal, N.; Attali, B. Targeting the Voltage Sensor of Kv7.2 Voltage-gated K+ Channels with a new Gating-modifier. *Proc. Natl. Acad. Sci. U.S.A.* **2010**, *107*, 15637–15642.

(6) Long, S.; Tao, X.; Campbell, E.; MacKinnon, R. Atomic Structure of a Voltage-dependent K+ channel in a Lipid Membrane-like Environment. *Nature.* **2007**, *450*, 376–82.

(7) Miceli, F.; Soldovieri, M. V.; Hernandez, C. C.; Shapiro, M. S.; Annunziato, L.; Taglialatela, M. Gating Consequences of Charge Neutralization of Arginine Residues in the S4 Segment of Kv7.2, an Epilepsy-Linked K+ Channel Subunit. *Biophys. J.* **2008**, *95*, 2254–2264.

(8) Miceli, F.; Soldovieri, M.; Ambrosino, P.; Barrese, V.; Migliore, M.; Cilio, M. R.;

Taglialatela, M. Genotype-phenotype Correlations in Neonatal Epilepsies Caused by Mutations in the Voltage Sensor of Kv7.2 Potassium Channel Subunits. *Proc. Natl. Acad. Sci. U.S.A.* **2013**, *110*, 4386–4391.

(9) Gourgy-Hacohen, O.; Kornilov, P.; Pittel, I.; Peretz, A.; Attali, B.; Paas, Y. Capturing Distinct KCNQ2 Channel Resting States by Metal Ion Bridges in the Voltage-sensor Domain. *J. Gen. Physiol.* **2014**, *144*, 513–527.

(10) Sun, J.; MacKinnon, R. Cryo-EM Structure of a KCNQ1/CaM Complex Reveals Insights into Congenital Long QT Syndrome. *Cell.* **2017**, *169*, 1042–1050.e9.

(11) Zimmermann, L.; Stephens, A.; Nam, S.-Z.; Rau, D.; Kübler, J.; Lozajic, M.; Gabler, F.; Söding, J.; Lupas, A. N.; Alva, V. A Completely Reimplemented MPI Bioinformatics Toolkit with a New HHpred Server at its Core. *J. Mol. Biol.* **2018**, *430*, 2237–2243.

(12) Söding, J.; Biegert, A.; Lupas, A. The HHpred Interactive Server for Protein Homology Detection and Structure Prediction. *Nucleic Acids Res.* **2005**, *33*, W244–8.

(13) Chys, P.; Chacón, P. Random Coordinate Descent with Spinor-matrices and Geometric Filters for Efficient Loop Closure. *J. Chem. Theory Comput.* **2013**, *9*, 1821–1829.

(14) López-Blanco, J. R.; Canosa-Valls, A. J.; Li, Y.; Chacón, P. RCD+: Fast Loop Modeling Server. *Nucleic Acids Res.* **2016**, *44*, W395–W400.

(15) Sun, J.; MacKinnon, R. Structural Basis of Human KCNQ1 Modulation and Gating. *Cell.* **2020**, *180*, 340–347.e9.

(16) Sievers, F.; Wilm, A.; Dineen, D.; Gibson, T. J.; Karplus, K.; Li, W.; Lopez, R.; McWilliam, H.; Remmert, M.; Söding, J.; Thompson, J. D.; Higgins, D. G. Fast, Scalable Generation of High-quality Protein Multiple Sequence Alignments using Clustal Omega. *Mol Syst Biol.* **2011**, *7*, 539.

(17) Waterhouse, A.; Rempfer, C.; Heer, F. T.; Studer, G.; Tauriello, G.; Bordoli, L.; Bertoni, M.; Gumienny, R.; Lepore, R.; Bienert, S.; de Beer, T. A.; Schwede, T. SWISS-MODEL: Homology Modelling of Protein Structures and Complexes. *Nucleic Acids Res.* **2018**, *46*, W296–W303.

(18) Sali, A.; Blundell, T. Comparative Protein Modelling by Satisfaction of Spatial Restraints. *J. Mol. Biol.* **1993**, *234*, 779–815.

(19) Pettersen, E. F.; Goddard, T. D.; Huang, C. C.; Couch, G. S.; Greenblatt, D. M.; Meng, E. C.; Ferrin, T. E. UCSF Chimera — A Visualization System for Exploratory Research and Analysis. *J. Comput. Chem.* **2004**, *25*, 1605–1612.

(20) Zhang, J.; Liang, Y.; Zhang, Y. Atomic-level Protein Structure Refinement Using Fragment-Guided Molecular Dynamics Conformation Sampling. *Structure.* **2011**, *19*, 1784–1795.

(21) Jo, S.; Kim, T.; Iyer, V.; Im, W. CHARMM-GUI: a Web-based Graphical User Interface for CHARMM. *J. Comput. Chem.* **2008**, *29*, 1859–1865.

(22) Jo, S.; Lim, J. B.; Klauda, J. B.; Im, W. CHARMM-GUI Membrane Builder for Mixed Bilayers and Its Application to Yeast Membranes. *Biophys. J.* **2009**, *97*, 50–58.

(23) Miceli, F.; Vargas, E.; Bezanilla, F.; Taglialatela, M. Gating Currents from Kv7 Channels Carrying Neuronal Hyperexcitability Mutations in the Voltage-Sensing Domain. *Biophys. J.* **2012**, *102*, 1372–1382.

(24) Miceli, F.; Soldovieri, M. V.; Ambrosino, P.; De Maria, M.; Migliore, M.; Migliore, R.; Taglialatela, M. Early-Onset Epileptic Encephalopathy Caused by Gain-of-Function Mutations in the Voltage Sensor of Kv7.2 and Kv7.3 Potassium Channel Subunits. *J. Neurosci.* **2015**, *35*, 3782–3793.

(25) Li, X.; Zhang, Q.; Guo, P.; Fu, J.; Mei, L.; Lv, D.; Wang, J.; Lai, D.; Ye, S.; Yang, H.; Guo, J. Molecular basis for ligand activation of the human KCNQ2 channel. *Cell. Res.*

(26) Jiang, D.; Gamal El-Din, T.; Ing, C.; Lu, P.; Pomès, R.; Zheng, N.; Catterall, W. Structural Basis for Gating Pore Current in Periodic Paralysis. *Nature.* **2018**, *557*, 590–594.

(27) Sadewa, A. H.; Sasongko, T. H.; ; Lee, M. J.; Daikoku, K.; Yamamoto, A.; Yamasaki, T.; Tanaka, S.; Matsuo, M.; Nishio, H. Germ-line Mutation of KCNQ2, p.R213W, in a Japanese Family with Benign Familial Neonatal Convulsion. *Pediatr Int.* **2008**, *50*, 167–171.

(28) Weckhuysen, S. et al. KCNQ2 Encephalopathy: Emerging Phenotype of a Neonatal Epileptic Encephalopathy. *Ann. Neurol.* **2012**, *71*, 15–25.

(29) Miceli, F.; Soldovieri, M.; Lugli, L.; Bellini, G.; Ambrosino, P.; Migliore, M.; Giudice, E.; Ferrari, F.; Pascotto, A.; Taglialatela, M. Neutralization of a Unique, Negatively-charged Residue in the Voltage Sensor of KV7.2 Subunits in a Sporadic Case of Benign Familial Neonatal Seizures. *Neurobiol. Dis.* **2009**, *34*, 501–510.

(30) Soldovieri, M.; Ambrosino, P.; Mosca, I.; Miceli, F.; Franco, C.; Canzoniero, L.; Kline-Fath, B.; Cooper, E.; Venkatesan, C.; Taglialatela, M. Epileptic Encephalopathy In A Patient With A Novel Variant In The Kv7.2 S2 Transmembrane Segment: Clinical, Genetic, and Functional Features. *Int. J. Mol. Sci.* **2019**, *20*, 3382.

(31) Maragliano, L.; Vanden-Eijnden, E. A Temperature Accelerated Method for Sampling Free Energy and Determining Reaction Pathways in Rare Events Simulations. *Chem. Phys. Lett.* **2006**, *426*, 168–175.

(32) Izrailev, S.; Stepaniants, S.; Balsera, M.; Oono, Y.; Schulten, K. Molecular Dynamics Study of Unbinding of the Avidin-biotin Complex. *Biophys. J.* **1997**, *72*, 1568–1581.

(33) Sotomayor, M.; Schulten, K. Single-Molecule Experiments in Vitro and in Silico. *Science.* **2007**, *316*, 1144–1148.

(34) De Fabritiis, G.; Coveney, P. V.; Villà-Freixa, J. Energetics of K+ Permeability through Gramicidin A by Forward-reverse Steered Molecular Dynamics. *Proteins.* **2008**, *73*, 185–194.
